# NKL homeobox gene activities in normal and malignant myeloid cells

**DOI:** 10.1371/journal.pone.0226212

**Published:** 2019-12-11

**Authors:** Stefan Nagel, Michaela Scherr, Roderick A. F. MacLeod, Claudia Pommerenke, Max Koeppel, Corinna Meyer, Maren Kaufmann, Iris Dallmann, Hans G. Drexler

**Affiliations:** 1 Department of Human and Animal Cell Lines, Leibniz-Institute DSMZ–German Collection of Microorganisms and Cell Cultures, Braunschweig, Germany; 2 Department of Hematology, Hemostasis, Oncology and Stem Cell Transplantation, Hannover Medical School, Hannover, Germany; European Institute of Oncology, ITALY

## Abstract

Recently, we have documented a hematopoietic NKL-code mapping physiological expression patterns of NKL homeobox genes in early hematopoiesis and in lymphopoiesis, which spotlights genes deregulated in lymphoid malignancies. Here, we enlarge this map to include normal NKL homeobox gene expressions in myelopoiesis by analyzing public expression profiling data and primary samples from developing and mature myeloid cells. We thus uncovered differential activities of six NKL homeobox genes, namely DLX2, HHEX, HLX, HMX1, NKX3-1 and VENTX. We further examined public expression profiling data of 251 acute myeloid leukemia (AML) and 183 myelodysplastic syndrome (MDS) patients, thereby identifying 24 deregulated genes. These results revealed frequent deregulation of NKL homeobox genes in myeloid malignancies. For detailed analysis we focused on NKL homeobox gene NANOG, which acts as a stem cell factor and is correspondingly expressed alone in hematopoietic progenitor cells. We detected aberrant expression of NANOG in a small subset of AML patients and in AML cell line NOMO-1, which served as a model. Karyotyping and genomic profiling discounted rearrangements of the NANOG locus at 12p13. But gene expression analyses of AML patients and AML cell lines after knockdown and overexpression of NANOG revealed regulators and target genes. Accordingly, NKL homeobox genes HHEX, DLX5 and DLX6, stem cell factors STAT3 and TET2, and the NOTCH-pathway were located upstream of NANOG while NKL homeobox genes HLX and VENTX, transcription factors KLF4 and MYB, and anti-apoptosis-factor MIR17HG represented target genes. In conclusion, we have extended the NKL-code to the myeloid lineage and thus identified several NKL homeobox genes deregulated in AML and MDS. These data indicate a common oncogenic role of NKL homeobox genes in both lymphoid and myeloid malignancies. For misexpressed NANOG we identified an aberrant regulatory network, which contributes to the understanding of the oncogenic activity of NKL homeobox genes.

## Introduction

Human hematopoiesis starts with hematopoietic stem/progenitor cells (HSPC) residing in specific niches in the bone marrow. These cells undergo self-renewal and generate lymphoid primed multipotent progenitors (LMPP), which supply both the lymphoid and myeloid lineage. Derived common lymphoid progenitors (CLP) and common myeloid progenitors (CMP) populate the entire casts of lymphocytes and myeloid blood cells, respectively [1]. The CMPs initiate the development of erythrocytes via the megakaryocytic-erythrocytic progenitor (MEP) and of granulocytes via the granulocyte-macrophage progenitor (GMP). Mature granulocytes comprise neutrophils, basophils and eosinophils, which differentiate via the transition stages of pro-myelocytes and meta-myelocytes. Additional myeloid blood cells are mast cells and monocytes the latter of, which are able to differentiate into dendritic cells in the bone marrow or into macrophages in non-hematopoietic tissues [2]. Of note, alternative hematopoietic models exist, which differ in the positioning and developmental potential of LMPPs and CMPs [3–5]. However, myeloid differentiation processes take place in the bone marrow and are prone to oncogenic alterations. Accordingly, chromosomal rearrangements, gene mutations, aberrant signalling pathways or alternative mechanisms drive the deregulation of particular developmental genes, which mediate an arrest of differentiation, enhanced survival and/or sustained proliferation [1,6,7].

Homeobox genes encode transcription factors (TFs), which regulate basic processes of cell, tissue, and organ differentiation in the embryo and in the adult [8,9]. The clustered HOX genes are expressed in a controlled pattern termed “HOX-code” which defines anterio-posterio organizations in embryonal structures such as that of the pharynx [10]. Accordingly, homeobox genes of the DLX group are expressed in the pharyngeal region in a dorso-ventral pattern organizing the embryonal pharynx complementary. This pattern has been termed “DLX-code” [11]. Recently, we coined the term hematopoietic “NKL-code” which describes the normal expression pattern of NKL homeobox genes in early hematopoiesis and in the lymphopoiesis including T-cell, B-cell and NK-cell development [12–14]. This homeobox gene subclass comprises 48 genes in humans–nine members included within the NKL-code hitherto comprising HHEX, HLX, MSX1, NANOG, NKX2-3, NKX3-1, NKX6-3, TLX2 and VENTX [12,15].

Deregulation of NKL homeobox genes is a frequent event in lymphoid malignancies instanced by 24 genes in T-cell leukemia, 19 in T-cell lymphoma, and 13 in B-cell lymphoma [12,13,16,17]. Thus, these genes represent a major group of lymphoid oncogenes. Aberrant activities of particular NKL homeobox genes have been described in myeloid malignancies as well, exemplified by DLX2, HLX and VENTX in acute myeloid leukemia (AML) [18–20]. However, a systematic analysis of NKL homeobox genes in normal myelopoiesis and myeloid malignancies is wanting hitherto. To fill this gap, we here screened public expression data to reveal physiological activities of NKL homeobox genes in myeloid development and mature myeloid blood cells. Additionally, we looked for aberrant expressions of NKL homeobox genes in patients of the most common myeloid malignancies namely AML and myelodyplastic syndrome (MDS). Finally, we focused on NKL homeobox gene NANOG and analyzed its aberrant activity in AML. The stem cell factor NANOG supports self-renewal and forms part of a basic gene network containing POU5F1/OCT4, SOX2, KLF4 and MYC (OSKM), which has been described in embryonal stem cells (ESCs) and is utilized to generate induced pluripotent stem cells (iPSCs) [21]. Our data uncovered an aberrant gene network around NANOG revealing deregulating upstream factors and potential oncogenic operations of this NKL homeobox gene in AML subsets.

## Materials and methods

### Transcriptome analysis by gene expression profiling and RNA sequencing

Public expression profiling datasets used in this study were obtained from Gene Expression Omnibus (GEO, www.ncbi.nlm.nih.gov) and generated by U133 Plus 2.0 gene chips from Affymetrix (High Wycombe, UK). Of note, this type of gene chip covers 37 of 48 known human NKL homeobox genes. We exploited datasets GSE42519, GSE22552, GSE109348 and GSE40831 for analyses of developing and mature myeloid cells [22,23]. Datasets GSE19429 and GSE15434 were analyzed for gene activities in MDS patients and AML patients with normal karyotype, respectively, and dataset GSE59808 to examine AML cell lines [24,25]. Dataset GSE61804 was generated from 325 AML patients with normal and abnormal karyotypes and GSE19577 from 42 AML patients with KMT2A translocations. To uncover potential biological functions of shortlisted genes, gene-annotation enrichment analysis was performed using DAVID bioinformatics resources (www.david.ncifcrf.org/) [26]. Expression profiling data of lentiviral-transfected HL-60 cells using HG U133 Plus 2.0 gene chip (Affymetrix) were generated by Dr. Robert Geffers (Genome Analytics Facility, Helmholtz Centre for Infection Research, Braunschweig, Germany). The primary data thus obtained are available at GEO via GSE131113. After RMA-background correction and quantile normalization of the spot intensities, the profiling data were expressed as ratios of sample means and subsequently log2 transformed. Data processing was performed via R/Bioconductor using public limma and affy packages.

Recently, we have sequenced polyA-enriched transcriptomes from 100 leukemia/lymphoma cell lines including ME-1, MOLM-13, MONO-MAC-6, MUTZ-3, THP-1 and U-937, which served as controls in this study [27]. Here, generation of transcriptome data of NOMO-1 was performed similarly, including RNA-extraction, quality control and sequencing protocol. These RNA sequencing data are deposited at ArrayExpress (www.ebi.ac.uk/arrayexpress) under accession numbers E-MTAB-7721 and E-MTAB-8010. Visualization of expression values was performed using the public R-package shinyngs.

### Polymerase chain-reaction (PCR) and Western blot analyses

Total RNA was extracted from cultivated cell lines using TRIzol reagent (Invitrogen, Darmstadt, Germany). Primary human RNA was obtained commercially. We used total RNA of mature granulocytes, CD14-positive monocytes, macrophages derived from monocytes by M-CSF stimulation and dendritic cells derived from monocytes by stimulation with a mix of cytokines, all obtained from 3H Biomedical AB (Uppsala, Sweden), human retina from Biochain (Newark, CA, USA), and CD34-positive HSPCs from Miltenyi Biotech (Bergisch-Gladbach, Germany). The derived cDNA was synthesized using 1 μg RNA, random priming, and Superscript II (Invitrogen), and was applied for PCR analyses.

Real-time quantitative (RQ)-PCR analyses were performed using the 7500 Real-time System, and commercial buffer and primer sets (Applied Biosystems/Life Technologies, Darmstadt, Germany). For normalization of expression levels we quantified the transcripts of TATA box binding protein (TBP). Quantitative analyses were performed in triplicate. Standard deviations in the figures are indicated as error bars. The statistical significance was assessed by Student´s t-test and the calculated p-values indicated by asterisks (* p<0.05, ** p<0.01, *** p<0.001, n.s. not significant).

Reverse-transcription (RT)-PCR analyses of fusion transcripts KMT2A-MLLT2 and KMT2A-MLLT3 were performed using the following oligonucleotides: KMT2A-F 5´-CAGGCACTTTGAACATCCTC-3´, MLLT2-rev 5´-CGTTCCTTGCTGAGAATTTG-3´, MLLT3-rev 5´-TACAGGCCTCTCCATTTCAG-3´. The gene YY1 served as control using the following oligonucleotides: YY1-for 5´-AAGCAGGTGCAGATCAAGAC-3´ and YY1-rev 5´-CCGAGTTATCCCTGAACATC-3´. All oligonucleotides were obtained from Eurofins MWG (Ebersberg, Germany). The PCR products were generated using taqpol (Qiagen, Hilden, Germany) and thermocycler TGradient (Biometra, Göttingen, Germany) and were subsequently analyzed by gel electrophoresis.

Western blot analysis was performed as described previously [16]. The following antibodies were used: alpha-Tubulin (Sigma), NANOG (Novus Biologicals, Abingdon, UK), KLF4 (Abcam, Cambridge, UK), phospho-STAT3 (Cell Signaling, Frankfurt, Germany), and TET2 (Boster Biological Technology, Pleasanton, CA).

### Cell lines and treatments

Cell lines were retrieved from the stocks of the Leibniz-Institute DSMZ (Braunschweig, Germany) and cultivated as described elsewhere [28]. For cytological analyses cell lines were stained with Giemsa-May-Grünwald as follows: cells were spun onto microscope slides or cultured in Clipmax chamber slides (TPP, Trasadingen, Switzerland) and fixed for 5 min with methanol. Subsequently they were stained for 3 min with May-Grünwald´s eosine-methylene blue solution modified (Merck, Darmstadt, Germany) diluted in Titrisol (Merck), and for 15 min with Giemsa´s azur eosin methylene blue solution (Merck). Images were captured with an AXIO Scope.A1 microscope using AxioCam MRc5 and software AxioVision 4.7 (Zeiss, Göttingen, Germany). Morphological cell alterations were analyzed after treatment for 5 days with 20 nM 12-O-Tetradecanoylphorbol 13-acetate (TPA) or 1.25% dimethyl sulfoxide (DMSO), both obtained from Sigma.

To manipulate gene expression levels we used gene specific siRNA oligonucleotides and AllStars negative Control siRNA (siCTR) obtained from Qiagen. A commercial gene expression construct for NANOG was cloned in vector pCMV6-XL5 and obtained from Origene (Wiesbaden, Germany). An empty vector served as control. SiRNAs (80 pmol) and expression constructs/vector controls (2 μg) were transfected into 1x10^6^ cells by electroporation using the EPI-2500 impulse generator (Fischer, Heidelberg, Germany) at 350 V for 10 ms. Electroporated cells were harvested after 20 h cultivation.

Pharmacological manipulations of gene activities were performed by treatments for 72 h with 20 nM TPA, for 20 h with 5 μM 3-Deazaneplanocin A (DZNep), 10 μg/ml trichostatin A (TSA), 100 μM AG490, 50 nM doxorubicine (doxo), 10 μM N-[N-(3,5-Difluorophenacetyl)-L-alanyl]-S-phenylglycine t-butyl ester (DAPT), and 10 μM dorsomorphin (DM) obtained from Sigma (Taufkirchen, Germany), or 10 μM IWR1 obtained from R&D Systems (Wiesbaden, Germany), all dissolved in DMSO. 5-Azacytidine (AZA) was also obtained from Sigma but dissolved in water and used at a final concentration of 50 μM. Additional manipulations were performed by treatments with 20 ng/ml M-CSF for 72 h or with 20 ng/ml BMP4 for 20 h (R&D Systems).

For functional studies treated cells were analyzed using the IncuCyte S3 Live-Cell Analysis System (Essen Bioscience, Hertfordshire, UK). Apoptosis was induced by treatments with 10 μM DAPT or 100 μM etoposide (Sigma) and detected via the IncuCyte Caspase-3/7 Green Apoptosis Assay diluted at 1:2000 (Essen Bioscience). Elongated cell morphology was quantified using the eccentricity tool. Analyses were performed in quadruplicates and calculated standard deviations were indicated in the figures as bars.

Forced NANOG expression in HL-60 cells was performed by lentiviral gene transfer. The plasmid pBShuttle-CMV-hNanog-WPRE containing the human NANOG gene (hNANOG) was kindly provided by Prof. Ulrich Martin (Hannover Medical School, Germany). The lentiviral vector pRRL.PPT.SF.i2GFP.pre used for transfection was kindly provided by Prof. Axel Schambach (Hannover Medical School, Germany). To create the lentiviral vector pRRL.ppt.SF.hNANOG.i2GFP.pre, the plasmid pRRL.PPT.SF.i2GFP.pre was digested with *BamH*I followed by a DNA Polymerase I fill-in reaction and subsequently gel-purified. The hNanog gene was isolated from the plasmid pBShuttle-CMV-hNANOG-WPRE using restriction enzymes *Xba*I and *Xho*I. The generated cohesive ends were then filled-in using Klenow enzyme. The obtained blunt-end fragment was about 980 bp long and ligated with the blunt-ended *BamH*I vector fragment, placing hNANOG downstream of the SFFV promoter. Preparation of recombinant lentiviral supernatants and lentiviral transductions were performed as described earlier [29]. Transduction efficacy into HL-60 cells was quantified by flow cytometric analysis based on GFP-expression at >98% in all experiments.

### Chromosomal and genomic analyses

Karyotyping and chromosomal analysis by fluorescence in situ hybridization (FISH) were performed as described previously [30]. BAC and fosmid clones were obtained from BacPac Resources (Children´s Hospital Oakland Research Institute, Oakland, CA, USA) to analyze the loci of BCR (RP11-164H20), ABL1 (RP11-142H20), and MIR17HG (G248P8960A3).

For genomic profiling of the cell lines NOMO-1 and K-562 their genomic DNA was prepared by the Qiagen Gentra Puregene Kit (Qiagen). Labelling, hybridization and scanning of Cytoscan HD arrays was performed at the Genome Analytics Facility, according to the manufacturer´s protocols (Affymetrix). Data were analyzed using the Chromosome Analysis Suite software version 3.1.0.15 (Affymetrix). Bioinformatic analysis of genome profiling data and whole transcriptome data was performed using public R-software and Excel (Microsoft, München, Germany). We extracted those genes from NOMO-1, which showed copy numbers higher or lower than two and connected them with corresponding expression values from RNA sequencing data of NOMO-1 and six AML control cell lines. A median was calculated for the expression values of the controls, which served as a baseline to identify enhanced or repressed genes in NOMO-1. Upregulated genes were defined by a factor of at least 1.5, downregulated genes by a factor of 0.7 (**S1 Table**).

## Results

### Normal and aberrant expression of NKL homeobox genes in myeloid cells

Recently, we reported the normal activities of NKL homeobox genes in early hematopoiesis and in lymphopoiesis, and termed these expression patterns the hematopoietic NKL-code [12–14]. Here, we analyzed the expression patterns of NKL homeobox genes in normal myelopoiesis. We screened public expression profiling data of myeloid progenitors and developmental stages of granulopoiesis and erythropoiesis (datasets GSE42519 and GSE22552), and of mature monocytes, mast cells and megakaryocytes (GSE109348, GSE40831). This exercise revealed activities of six NKL homeobox genes in normal myelopoiesis, including DLX2, HHEX, HLX, HMX1, NKX3-1 and VENTX (**S1 Fig**). While HHEX, HLX and VENTX were expressed in most samples HMX1 expression was restricted to the erythropoietic lineage, DLX2 to mature mast cells and monocytes, and NKX3-1 to mature granulocytes and monocytes.

**Fig 1:**
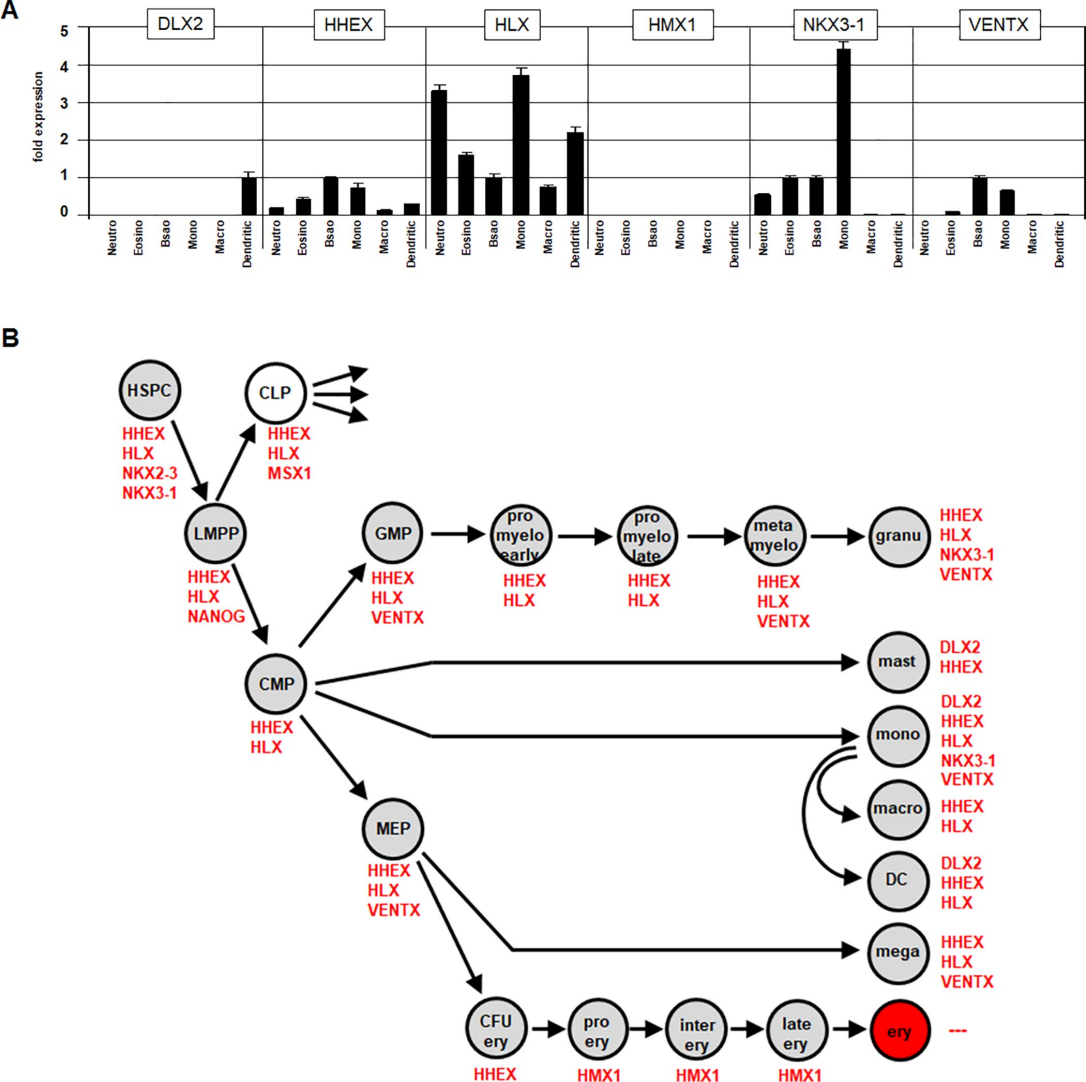
Creation of the myeloid NKL-code. (A) Expression values of NKL homeobox genes DLX2, HHEX, HLX, HMX1, NKX3-1 and VENTX were quantified by RQ-PCR analysis in primary blood cell samples from neutrophils, eosinophils, basophils, monocytes, macrophages and dendritic cells. For clarity the data are given in relation to basophils (except for DLX2) and set to 1. (B) This schematic presentation of active NKL homeobox genes in developing and mature myeloid cells displays the myeloid NKL-code.

Furthermore, we performed RQ-PCR analysis of normal primary myeloid cells to quantify DLX2, HHEX, HLX, HMX1, NKX3-1 and VENTX expression in mature subtypes of granulocytes including neutrophils, eosinophils and basophils, in addition to mature monocytes, macrophages and dendritic cells (**Fig 1A**). HHEX and HLX expression was detected in all samples analyzed while HMX1 was absent throughout. Concerning DLX2, the detection of positive expression just in dendritic cells belied the profiling data, which indicated positive expression values in mast cells and monocytes. However, significant expression levels were detected for NKX3-1 in neutrophils, eosinophils, basophils and monocytes, and for VENTX in basophils and monocytes. The comparison of expression data from monocytes with those from monocyte-derived macrophages and dendritic cells demonstrated downregulation of HHEX, HLX, NKX3-1 and VENTX in both cell types. In dendritic cells we detected DLX2 expression while monocytes tested negative. Thus, these data showed dynamic gene activities indicating their functional relevance for monocytic differentiation processes. Finally, we have combined the results obtained from expression profiling and RQ-PCR analysis to infer a myeloid NKL-code as shown in **Table 1** and **Fig 1B**.

**Table 1:** NKL homeobox gene activities in normal myelopoiesis and myeloid malignancies.

Gene	HSC	LMPP	CMP	GMP	PM	MM	G	Mas	Mo	Mac	Meg	PE	E	AML	MDS
**DLX2**								+	+					+	+
**HHEX**	+	+	+	+	+	+	+	+	+	+	+			+	+
**HLX**	+	+	+	+	+	+	+		+	+	+			+	+
**HMX1**												+			+
**MSX1**														+	+
**NANOG**		+												+	+
**NKX2-3**	+														+
**NKX3-1**	+						+		+					+	+
**NKX6-3**															+
**VENTX**				+		+	+		+		+			+	+
**BARHL1**															+
**BARX1**														+	
**DLX1**														+	+
**DLX3**														+	
**DLX4**														+	
**DLX6**														+	
**EMX1**															+
**EMX2**														+	
**HMX2**														+	
**MSX2**														+	
**NKX1-1**															+
**NKX2-5**														+	
**NKX3-2**														+	
**VAX2**														+	
24	4	3	2	3	2	3	4	2	5	2	3	1	0	18	14

HSC: hematopoietic stem cell, LMPP: lymphoid primed multipotent progenitor, CMP: common myeloid progenitor, GMP: granulocyte macrophage progenitor, PM: pro-myelocyte, MM: meta-myelocyte, G: granulocyte, Mas: mast cell, Mo: monocyte, Mac: macrophage, Meg: megakaryocyte, PE: pro-erythrocyte, E: erythrocyte, AML: acute myeloid leukemia, MDS: myelodysplastic syndrome. (+) indicates detected gene activity in the corresponding entity.

To identify aberrantly expressed NKL homeobox genes in myeloid malignancies we screened public expression profiling data of 251 AML and 183 MDS patient samples using datasets GSE15434 (**S2 Fig**) and GSE19429 (**S3 Fig**), respectively. This examination revealed 18 deregulated NKL homeobox genes in AML patients with normal karyotype and 14 in MDS patients (**Table 1**). Together, we found in these AML and MDS patients 24 deregulated NKL homeobox genes, including BARHL1, BARX1, DLX1, DLX2, DLX3, DLX4, DLX6, EMX1, EMX2, HHEX, HLX, HMX1, HMX2, MSX1, MSX2, NANOG, NKX1-1, NKX2-3, NKX2-5, NKX3-1, NKX3-2, NKX6-3, VAX2 and VENTX. Of note, 10/24 (42%) of these genes belong to the extended hematopoietic NKL-code while the remaining 14/24 (58%) ones were ectopically expressed. Furthermore, screening of public expression profiling data for 32 AML cell lines (GSE59808) showed deregulation of 26 NKL homeobox genes (**S4 Fig**), revealing suitable models to study particular genes in this disease.

Thus, we have identified normal NKL homeobox gene activities in myelopoiesis and aberrantly expressed genes in AML and MDS patients, which include overexpressed NKL-code members and ectopically activated non-code members. These data show that deregulated NKL homeobox genes play a significant role in myeloid malignancies as well.

### Aberrant expression of NANOG in AML

NKL homeobox genes encode TFs which control physiological differentiation processes in hematopoietic cells and tissues. When deregulated they might disturb these processes generating malignant cells. Therefore, we subsequently focused on NKL homeobox gene NANOG, which represses cell differentiation in early embryogenesis as well as in different types of stem cells in the adult, and has been correspondingly detected in LMPPs [12,21]. Our data showed aberrant overexpression in 2% AML patients with normal karyotype (**S2 Fig**), indicating NANOG as a surprisingly rarely activated oncogene in this malignancy. Analysis of dataset GSE61804 which contains 325 AML samples with normal and abnormal karyotypes indicated equally infrequent NANOG-overexpressing patients (11/325, 3.4%), unrelated to particular chromosomal aberrations thus excluding subtype-specific activity (**S5 Fig**).

To reveal upstream and downstream gene candidates of NANOG in AML we performed comparative expression analysis of public profiling data from AML patients with normal karyotype (**S6A Fig**) and AML cell lines (**S6B Fig**). We used the GEO-associated online-tool GEO2R, which calculates the top-250 most significant differentially expressed genes in selected NANOG-high and NANOG-low samples. Subsequent gene-annotation enrichment analysis of these genes highlighted gene ontology (GO)-terms denoting transcription (p = 6.9x10^-6^), cell differentiation (p = 4.1x10^-2^), and apoptosis (p = 5.9x10^-2^) for the AML patients (**S6A Fig**), and GO-terms apoptosis (p = 2.8x10^-5^), cell cycle (p = 3.0x10^-2^), and NOTCH-signalling (p = 7.7x10^-2^) for the AML cell lines (**S6B Fig**). Selected genes from both analyses were classified into five categories including chromatin (BCL7B, BRD2, EHMT2, HDAC7, JADE2, KDM1A, KDM3B, RNF7), transcription factors (MYBL1, MYC, STAT3, TLE4), BMP- and NOTCH-signalling pathways (BMP1, BMPR2, MAML2), proliferation (CDK11B, CCND1, SLFN13), and apoptosis (BCL2, MCL1). These identified categories/genes are reported players in leukemogenesis and might be regulatory connected with the aberrant activity of NANOG.

### Expression and mutual regulation of NKL-code members in AML cell lines

**Fig 2:**
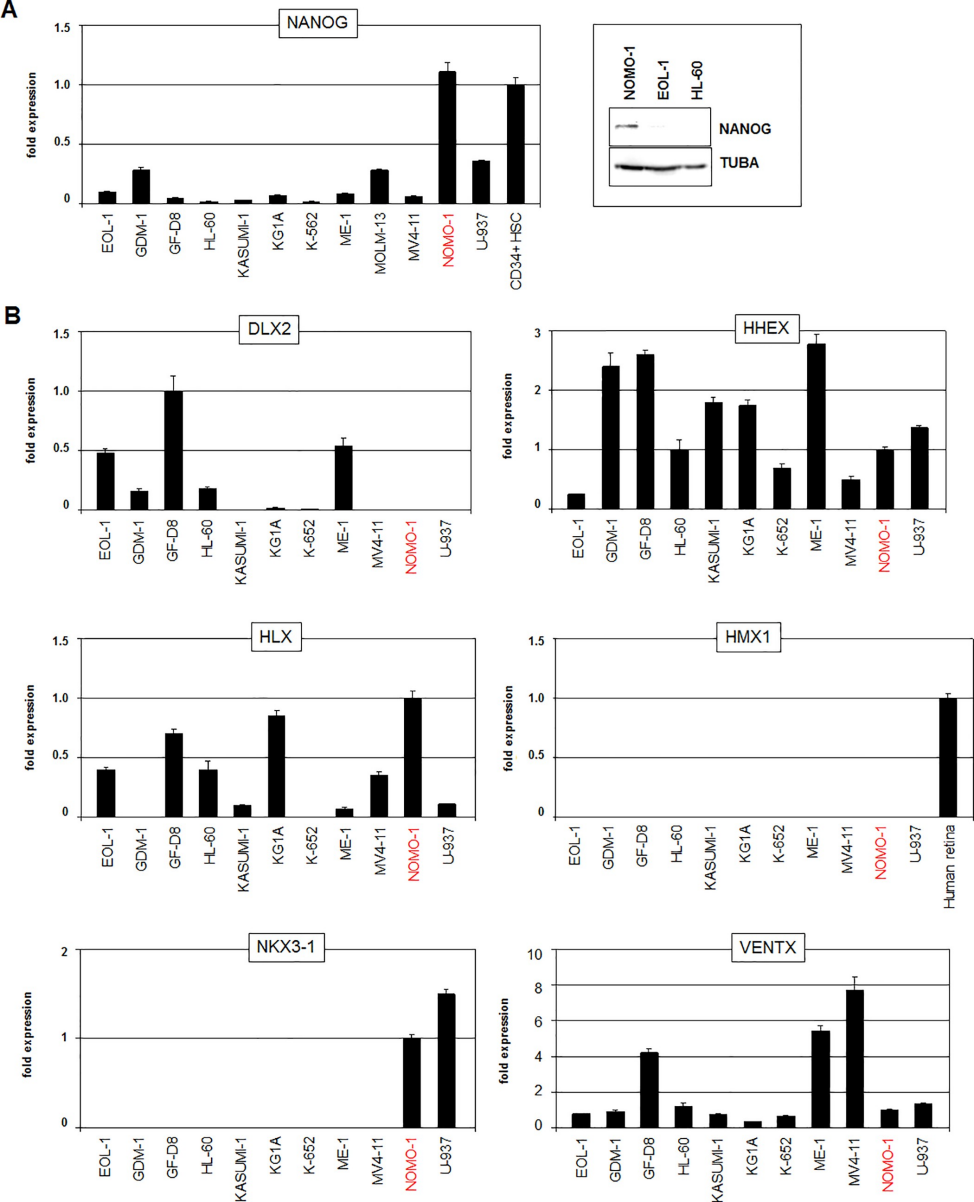
NKL homeobox gene expression in myeloid cell lines. (A) RQ-PCR analysis (left) and western blot analysis (right) of NANOG demonstrate enhanced expression levels in cell line NOMO-1 and in primary hematopoietic stem cells the level of, which was set to 1. TUBA served as loading control. (B) RQ-PCR analysis of six myeloid NKL-code members in eleven myeloid cell lines. A sample from human retina served as positive control for analysis of HMX1.

Our screening approach using public expression profiling data from AML cell lines demonstrated overexpression of NANOG in NOMO-1 (**S4 Fig**). RQ-PCR and western blot analyses of selected myeloid cell lines confirmed enhanced NANOG expression therein (**Fig 2A**). Moreover, the transcript level of stem cell factor NANOG in NOMO-1 matched that quantified in a primary CD34-positive hematopoietic stem cell sample (**Fig 2A**), evidencing significant expression of NANOG in this cell line. Therefore, NOMO-1 was chosen as a model for functional analyses of deregulating mechanisms and target genes of NANOG in AML.

**Fig 3:**
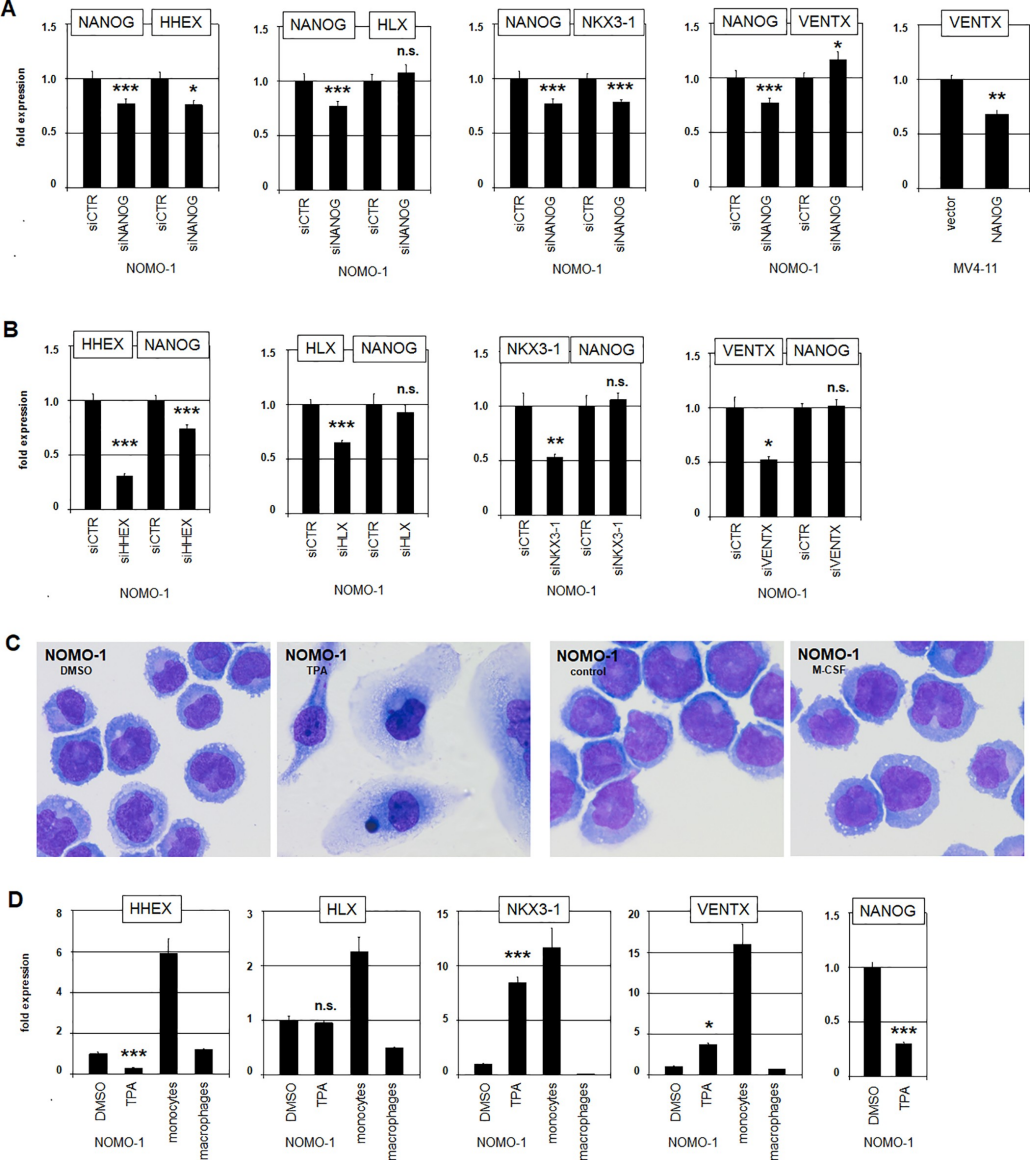
Regulation of myeloid NKL homeobox genes. (A) RQ-PCR analysis of NOMO-1 after siRNA-mediated knockdown of NANOG for HHEX, HLX, NKX3-1 and VENTX. RQ-PCR analysis of MV4-11 after forced expression of NANOG for VENTX (right). These data demonstrate regulatory impacts of NANOG on HHEX, NKX3-1 and VENTX. (B) RQ-PCR analysis of NANOG in NOMO-1 after siRNA-mediated knockdown of HHEX, HLX, NKX3-1 and VENTX. Just HHEX activates the expression of NANOG. (C) Giemsa-staining of NOMO-1 cells after treatment with TPA (left) and M-CSF (right). DMSO and medium controls are shown aside. (D) RQ-PCR analysis of NKL homeobox genes HHEX, HLX, NKX3-1, VENTX and NANOG in NOMO-1 after treatment with TPA in comparison to primary cell samples of monocytes and macrophages. Levels of the DMSO-controls were set to 1.

RQ-PCR analysis of the myeloid NKL-code members DLX2, HHEX, HLX, HMX1, NKX3-1 and VENTX was performed in NOMO-1 in addition to 10 myeloid control cell lines and selected primary cell/tissue samples (**Fig 2B**). These data showed that NOMO-1 also expressed elevated HHEX, HLX and NKX3-1 while the expression of VENTX resembled most controls at a lower level and DLX2 and HMX1 were silenced. To analyze potential regulatory relationships between NANOG and co-expressed NKL-code members we performed knockdown and overexpression experiments. SiRNA-mediated knockdown of NANOG in NOMO-1 indicated that NANOG activated the expression of HHEX and NKX3-1, and (slightly) inhibited VENTX while HLX was not regulated (**Fig 3A**). Forced expression of NANOG in low-expressing MV4-11 cells via electroporation of a corresponding gene construct resulted in decreased VENTX levels (**Fig 3A**), supporting a repressive impact for NANOG on this gene. SiRNA-mediated knockdown of HHEX in NOMO-1 resulted in reduced expression levels of NANOG (**Fig 3B**), indicating mutual activation of these genes. Knockdowns of HLX, NKX3-1 or VENTX left NANOG expression levels unperturbed (**Fig 3B**), demonstrating absence of regulation. Thus, NANOG is part of a regulatory gene network comprising the myeloid NKL-code members HHEX, NKX3-1 and VENTX. This network may play a role in normal stem/progenitor cells as well while aberrant activity of NANOG may deregulate these genes in malignant myeloid cells in patients.

NOMO-1 represents an immature monocytic cell line, which has been shown to differentiate after stimulation with TPA [28,31]. Accordingly, after three days of treatment we observed altered cytological characteristics including adherence, flattening and formation of spikes (**Fig 3C**). RQ-PCR analysis of NKL homeobox genes in these cells demonstrated that TPA-treatment resulted in downregulation of HHEX and NANOG, upregulation of NKX3-1 and VENTX while HLX remained unchanged (**Fig 3D**). M-CSF-signalling is involved in the differentiation of primary monocytes into macrophages [32]. To simulate this process we treated NOMO-1 cells with the ligand M-CSF for three days. However, these cells displayed no cytological alterations (**Fig 3C**). In addition, RQ-PCR analysis demonstrated just insignificant changes. Together, these data showed altered expression levels of particular myeloid NKL-code members in the course of TPA-induced monocytic cell differentiation, supporting a potential role for their physiological activities in developmental processes. Moreover, these processes were open to influence by NANOG in the malignant context.

### Karyotype and genomic profiling of NOMO-1

**Fig 4:**
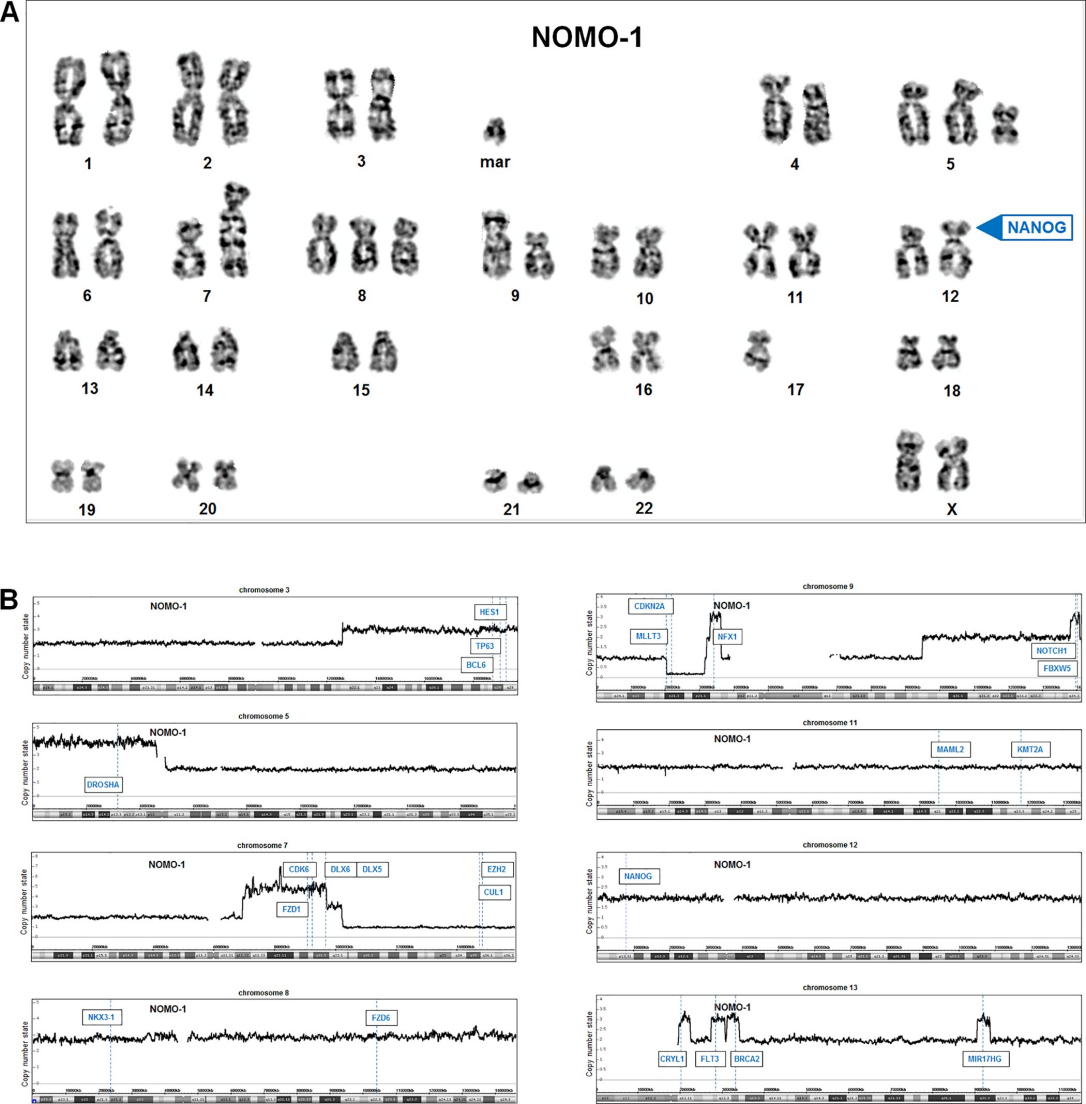
Karyotype and genomic profiling of NOMO-1. (A) Karyogram of NOMO-1 showing an aberrant karyotype as described in the text. The locus of NANOG at 12p13 is indicated, showing absence of chromosomal aberrations. (B) Genomic profiling of NOMO-1 indicated copy number alterations in the chromosomes 3, 5, 7, 8, 9, 11, 12, and 13. Selected genes are indicated.

To look for genomic alterations underlying aberrant activation of NANOG in NOMO-1 cells we performed karyotyping and genomic profiling. Examinations of generated karyograms revealed the following karyotype for this cell line: 46–48<2n>XX, +5, i(5)(p10), add(7)(q32), +8, der(9)dup(9)(p11p13)del(9)(p13-p21), t(9;11)(p22;q23)add(9)(q13), ins(13;?)(q12), +mar (**Fig 4A**). This result demonstrated normal chromosomal configurations at 12p13, indicating absence of rearrangements of NANOG, which is located at this position. Nevertheless, karyotype analysis confirmed the reported aberration t(9;11)(p22;q23), which generates the fusion gene KMT2A-MLLT3 [33].

Furthermore, genomic profiling data showed gains at 3q22-q29, at 5p corresponding to i(5)(p10), at 7q11-q22, of the complete chromosome 8, at 9p13-p21, 9q34, 13q12 and 13q21, and deletions at 7q31-q36 and 9p21. However, these data demonstrated absence of amplifications at 12p13 (**Fig 4B**). Thus, karyotyping and copy number analysis excluded activating alterations at the locus of NANOG in NOMO-1.

### Identification of NANOG-associated genes in NOMO-1

Our chromosomal and genomic data indicated several translocated, amplified and deleted genes, which might be indirectly connected with aberrant NANOG activity in NOMO-1, to serve as candidate regulators or targets (**Fig 4**). To identify genes activated or repressed by copy number alterations we combined our genomic profiling data with RNA sequencing data generated for NOMO-1 and six published controls comprising AML cell lines ME-1, MOLM-13, MONO-MAC-6, MUTZ-3, THP-1 and U-937 (**S1 Table**). This procedure revealed a total number of 1087 deregulated genes in NOMO-1. These genes were subsequently used for gene-annotation enrichment analysis and indicated several GO-terms including apoptotic processes (p = 5.2x10^-3^), NOTCH signalling (p = 5.3x10^-3^), WNT-signalling (p = 8.8x10^-3^) and mitotic cell cycle (p = 4.9x10^-2^).

**Fig 5:**
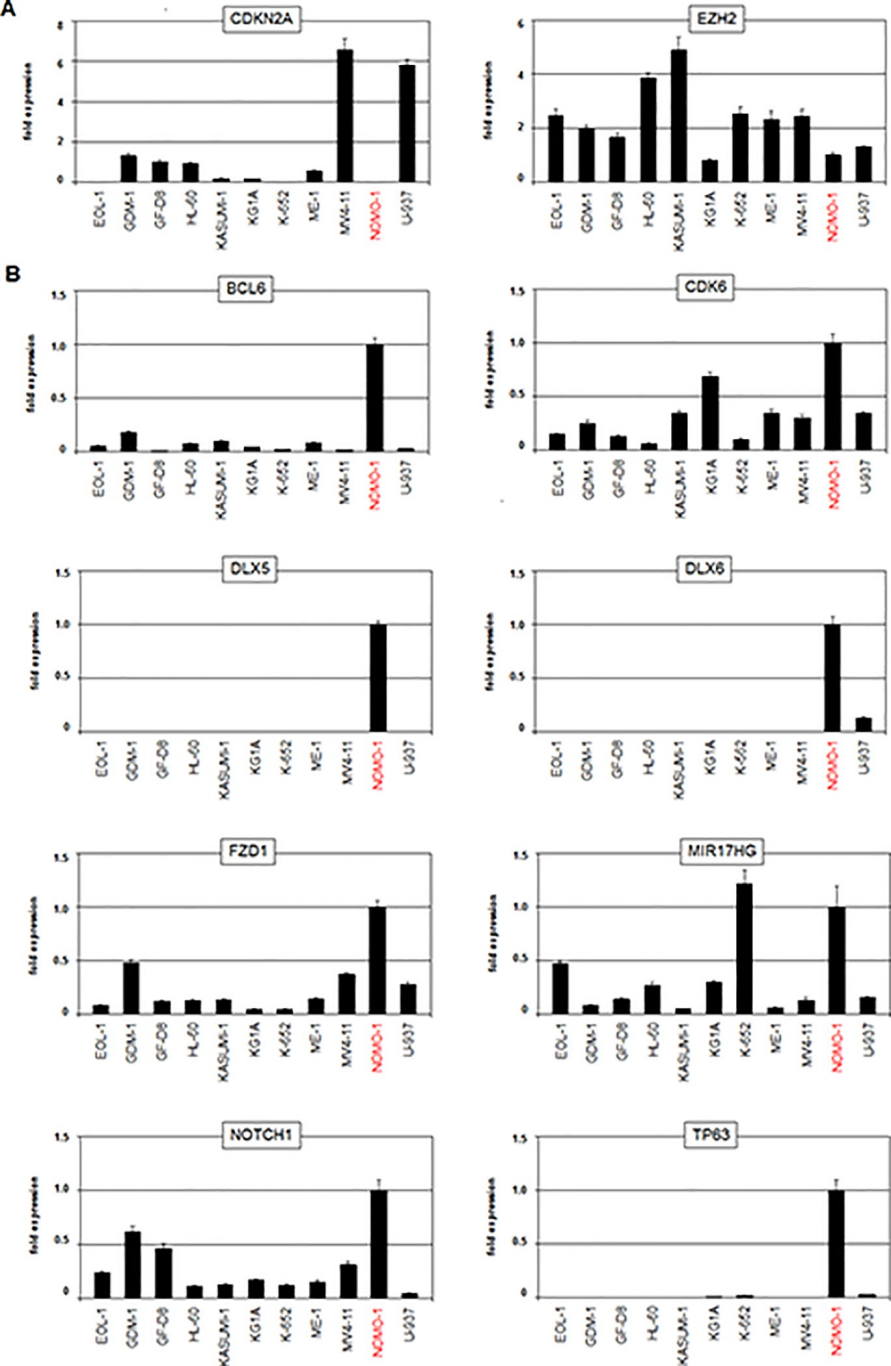
Expression analyses of candidate genes in AML cell lines. (A) RQ-PCR analysis of candidate genes showing copy number loss in NOMO-1 including CDKN2A and EZH2. (B) RQ-PCR analysis of candidate genes showing copy number gains in NOMO-1 including BCL6, CDK6, DLX5, DLX6, FZD1, MIR17HG, NOTCH1 and TP63. The expression levels from NOMO-1 were set to 1.

Next we selected from this group of genes potential NANOG-associated candidates and classified these into functional categories including chromatin (BRCA2, EZH2, KMT2A, MLLT3), TFs (BCL6, BHLHE22, DLX5, DLX6, NKX3-1, TP63), NOTCH- and WNT-signalling pathways (CUL1, FBXW5, FZD1, FZD6, HES1, NFX1, NOTCH1), proliferation (CDK6, CDKN2A), and micro-RNA/apoptosis (DROSHA, MIR17HG). RQ-PCR analysis of 10 gene candidates in myeloid cell lines confirmed downregulation of CDKN2A and EZH2 (**Fig 5A**) and upregulation of BCL6, CDK6, DLX5, DLX6, FZD1, MIR17HG, NOTCH1 and TP63 in NOMO-1 (**Fig 5B**).

Conspicuous coincidences of categories identified via this genomic approach with those from our comparative expression profiling of patients and cell lines (see above and **S6 Fig**) prompted more detailed examination of potential NANOG activators, including selected chromatin and transcription factors, and NOTCH-, WNT- and BMP-pathways.

### NOTCH-signalling is located upstream of NANOG

**Fig 6:**
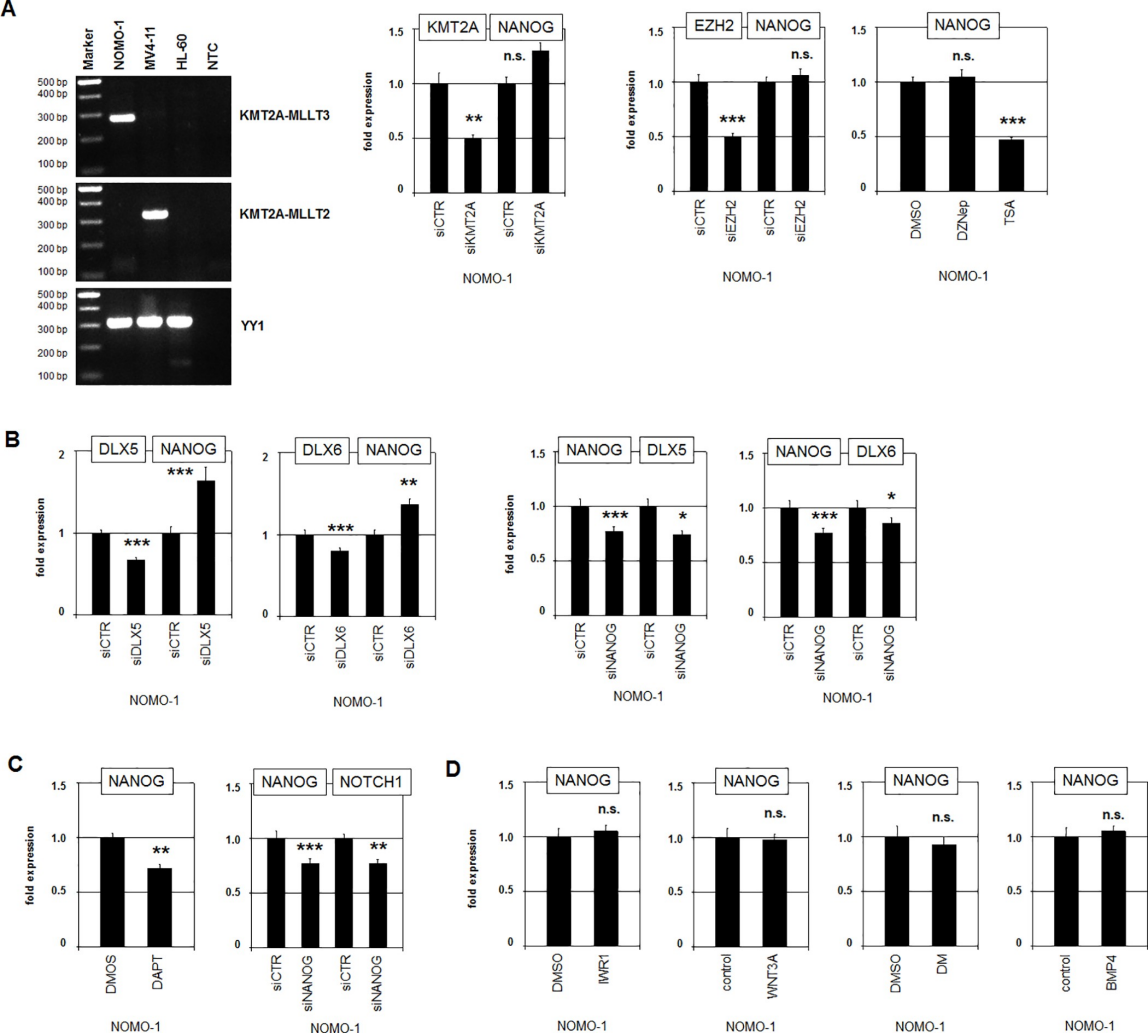
Analyses of chromatin factors and signalling pathways on NANOG. (A) RT-PCR analysis of fusion gene transcripts in AML cell lines demonstrating the presence of KMT2A-MLLT3 in NOMO-1 and of KMT2A-MLLT2 in MV4-11. YY1 served as cDNA-control and HL-60 as negative control; NTC: no template control (left). RQ-PCR analysis of NANOG in NOMO-1 cells after siRNA-mediated knockdown of KMT2A and EZH2 and after treatment with EZH2-inhibitor DZNep and HDAC-inhibitor TSA (right). (B) RQ-PCR analysis of NOMO-1 cells after siRNA-mediated knockdown of DLX5, DLX6 and NANOG. (C) RQ-PCR analysis of NOMO-1 cells after treatment with NOTCH-inhibitor DAPT (left) and after siRNA-mediated knockdown of NANOG (right), indicating mutual activation of NOTCH-signalling and NANOG. (D) RQ-PCR analysis of NANOG in NOMO-1 cells treated with WNT-inhibitor IWR1, WNT-ligand WNT3A, BMP-receptor-inhibitor dorsomorphin (DM) and BMP-ligand BMP4. These treatments indicated absence of regulatory impacts.

KMT2A/MLL encodes a histone methyltransferase, which generates active chromatin marks. KMT2A fusion genes mediate aberrant gene activation in AML subsets and KMT2A-MLLT2 formation has been shown to correlate with activated NANOG [34,35]. The presence of fusion transcript KMT2A-MLLT2 was confirmed by RT-PCR in MV4-11 and that of KMT2A-MLLT3 in NOMO-1 cells (**Fig 6A**). However, siRNA-mediated knockdown of KMT2A in NOMO-1 failed to inhibit NANOG expression (**Fig 6A**), demonstrating absence of any regulatory impact. EZH2 encodes the enzymatic core of polycomb repressor complex (PRC)2, which counteracts KMT2A and suppresses several developmental genes including NKL subclass members [16,36]. However, siRNA-mediated knockdown or pharmacological inhibition of EZH2 by DZNep also failed to alter NANOG expression levels (**Fig 6A**). In contrast, treatment of NOMO-1 cells with histone deacetylase-inhibitor TSA resulted in reduced NANOG expression (**Fig 6A**). Acetylated histones decompact the chromatin and are connected with gene activity. Therefore, decreased transcription of NANOG after TSA treatment indicated that histone acetylation played no significant role in NANOG regulation. In fact, these data suggested a decisive input of an other TF regulated by acetylation.

DLX5 and DLX6 encode transcription factors that are also members of the NKL homeobox gene subclass but normally expressed in neural crest cells [15,37]. Their genes are arranged in tandem and located within a duplicated region at 7q21. Surprisingly, siRNA-mediated knockdown of both DLX5 and DLX6 resulted in increased NANOG expression levels (**Fig 6B**), indicating an inhibitory effect. In contrast, knockdown of NANOG resulted in decreased expression levels of both DLX5 and DLX6 (**Fig 6B**). Thus, NANOG activated DLX5 and DLX6 which in turn reduced the NANOG transcript levels, generating a negative feedback-system.

NOTCH1 is located in an amplicon at 9q34 and encodes a receptor activated by ligand binding and subsequent proteolytic release of its intracellular part [38]. Pharmacological inhibition of this NOTCH-cleavage by DAPT resulted in decreased transcript levels of NANOG (**Fig 6C**), indicating that NOTCH-signalling activated NANOG transcription. Moreover, siRNA-mediated knockdown of NANOG resulted in decreased expression of NOTCH1 (**Fig 6C**), demonstrating mutual activation of NOTCH-signalling and NANOG. Functional analysis of the NOTCH-pathway was performed by live-cell-imaging. Accordingly, treatment of NOMO-1 cells with NOTCH-inhibitor DAPT spared cell proliferation while caspase-mediated apoptosis was strongly enhanced (**S7A Fig**). Together, these data indicated that the NOTCH-pathway together with NANOG generated a positive feedback-system, which supports survival in this AML cell line.

FZD1 and FZD6 encode receptors of the WNT-signalling pathway, which has been shown to regulate NANOG expression in stem cells [39,40]. Both genes were overexpressed in NOMO-1 probably via chromosomal gains at 7q21 and 8q22, respectively. However, pharmacological inhibition of WNT-signalling by IWR1 or stimulation with recombinant ligand WNT3A showed no change of NANOG expression in NOMO-1 (**Fig 6D**). Similarly, a potential impact of the BMP-pathway on NANOG expression was analyzed in NOMO-1 cells by treatment with BMP-receptor inhibitor dorsomorphin (DM) and activating ligand BMP4. However, these stimulations also failed to alter NANOG expression (**Fig 6D**). Thus, WNT- and BMP-signalling presumably played no role in the deregulation of NANOG in NOMO-1 cells.

### Stem cell factors STAT3 and TET2 activate NANOG in NOMO-1

**Fig 7:**
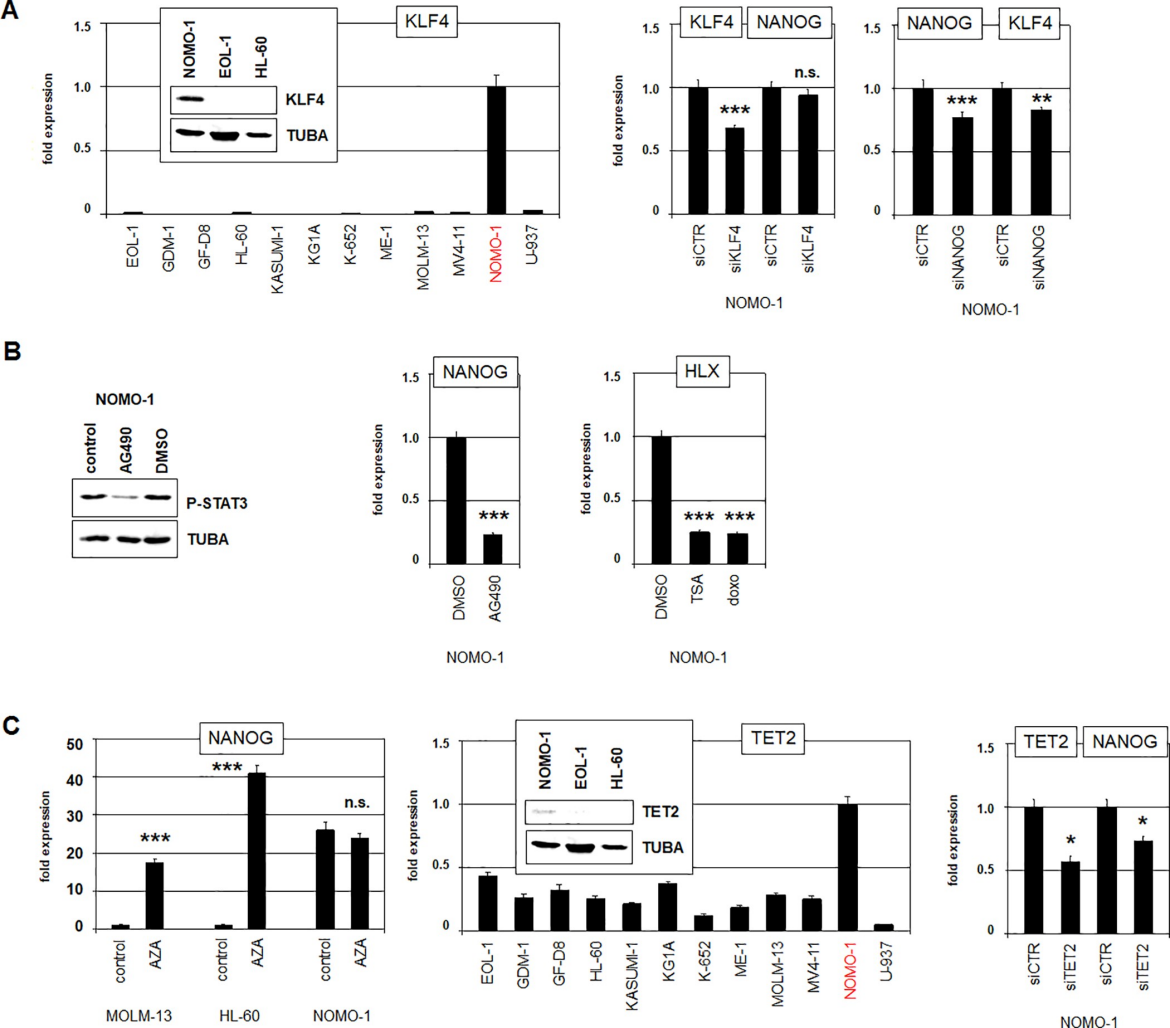
Regulatory relationships between KLF4, STAT3, TET2 and NANOG. (A) RQ-PCR analysis of KLF4 in myeloid cell lines (left) and western blot analysis of KLF4 (insert). RQ-PCR analysis of NOMO-1 cells after siRNA-mediated knockdown of KLF4 (middle), and NANOG (right), showing that KLF4 is a target of NANOG. (B) Western blot analysis of phospho-STAT3 in NOMO-1 cells treated with STAT3-inhibitor AG490 (left). RQ-PCR analysis of NANOG in NOMO-1 treated with AG490 (middle). RQ-PCR analysis of HLX in NOMO-1 treated with TSA and doxorubicine (right). (C) RQ-PCR analysis of NANOG in AML cell lines MOLM-13, HL-60 and NOMO-1 after treatment with AZA (left). RQ-PCR analysis of TET2 in myeloid cell lines (middle) and western blot analysis of TET2 (insert). RQ-PCR analysis of TET2 and NANOG in NOMO-1 after siRNA-mediated knockdown of TET2 (right).

NANOG is closely associated to a gene regulatory network described in ESCs and iPSCs comprising four transcription factors namely POU5F1/OCT4, SOX2, KLF4 and MYC [21]. To examine the potential impact of these factors on NANOG activation in AML we first determined their expression levels in NOMO-1 in comparison to myeloid control cell lines by RNA-sequencing (**S8A Fig),** RQ-PCR and western blot (**Fig 7A**). These data demonstrated conspicuously enhanced activity of KLF4 in NOMO-1 cells while the remaining transcription factors were absent or insignificantly expressed. However, siRNA-mediated knockdown of KLF4 in NOMO-1 failed to reduce the expression of NANOG (**Fig 7A**), indicating that stem cell factor KLF4 did not activate transcription of NANOG in NOMO-1 as reported for ESCs [41]. In contrast, siRNA-mediated knockdown of NANOG resulted in reduced expression of KLF4 (**Fig 7A**), showing that KLF4 is a target of NANOG in NOMO-1.

In addition to the reportedly mutual regulation of NANOG and the OSKM-factors, NANOG is activated in stem cells by STAT3 [42]. Accordingly, treatment of NOMO-1 with STAT3-inhibitor AG490 resulted in reduced STAT3-phosphorylation and concomitant downregulation of NANOG (**Fig 7B**), indicating that STAT3 activated NANOG in this AML cell line as well. In lymphoid cells acetylation of STAT3 mediates nuclear export and thus suppression of its target gene HLX [43,44]. Therefore, our observation of decreased NANOG expression in NOMO-1 cells after treatment with deacetylase-inhibitor TSA as shown in **Fig 6A** suggested that enhanced acetylation of STAT3 might be also responsible for this effect in myeloid cells. Accordingly, RQ-PCR analysis of NOMO-1 demonstrated decreased expression of HLX after treatment with TSA and STAT3-inhibitor doxorubicine as well (**Fig 7B**), indicating that both NANOG and HLX are regulated by STAT3 in this AML cell line.

In the course of stem cell differentiation the gene activity of NANOG is repressed by DNA-methylation of its promoter region [45]. To investigate this mode of regulation in AML we treated NANOG-low cell lines MOLM-13 and HL-60 with DNA-methylation-inhibitor AZA. This stimulation resulted in strongly increased expression levels of NANOG (**Fig 7C**), supporting that DNA-methylation plays a significant role for the suppression of NANOG in AML cells as well. In NOMO-1 cells the level of NANOG remained constantly high after AZA-treatment (**Fig 7C**), indicating that the critical loss of DNA-demethylation required for maximal NANOG activation has already been accomplished.

But why is the NANOG gene demethylated/activated in NOMO-1? RNA-sequencing data and subsequent RQ-PCR and western blot analyses demonstrated elevated expression levels of DNA-demethylating factor TET2 exclusively in NOMO-1 while other factors involved in DNA-methylation analyzed were inconspicuously expressed (**Figs S8B** and **7C**). Consistent with this picture, siRNA-mediated knockdown of TET2 in NOMO-1 reduced the expression level of NANOG (**Fig 7C**), demonstrating an activating impact. Taken together, these data indicated that aberrantly overexpressed TET2 reduced DNA-methylation, which in turn supported enhanced expression of NANOG in AML cell line NOMO-1.

### Target gene analysis of NANOG in transduced HL-60 cells

**Fig 8:**
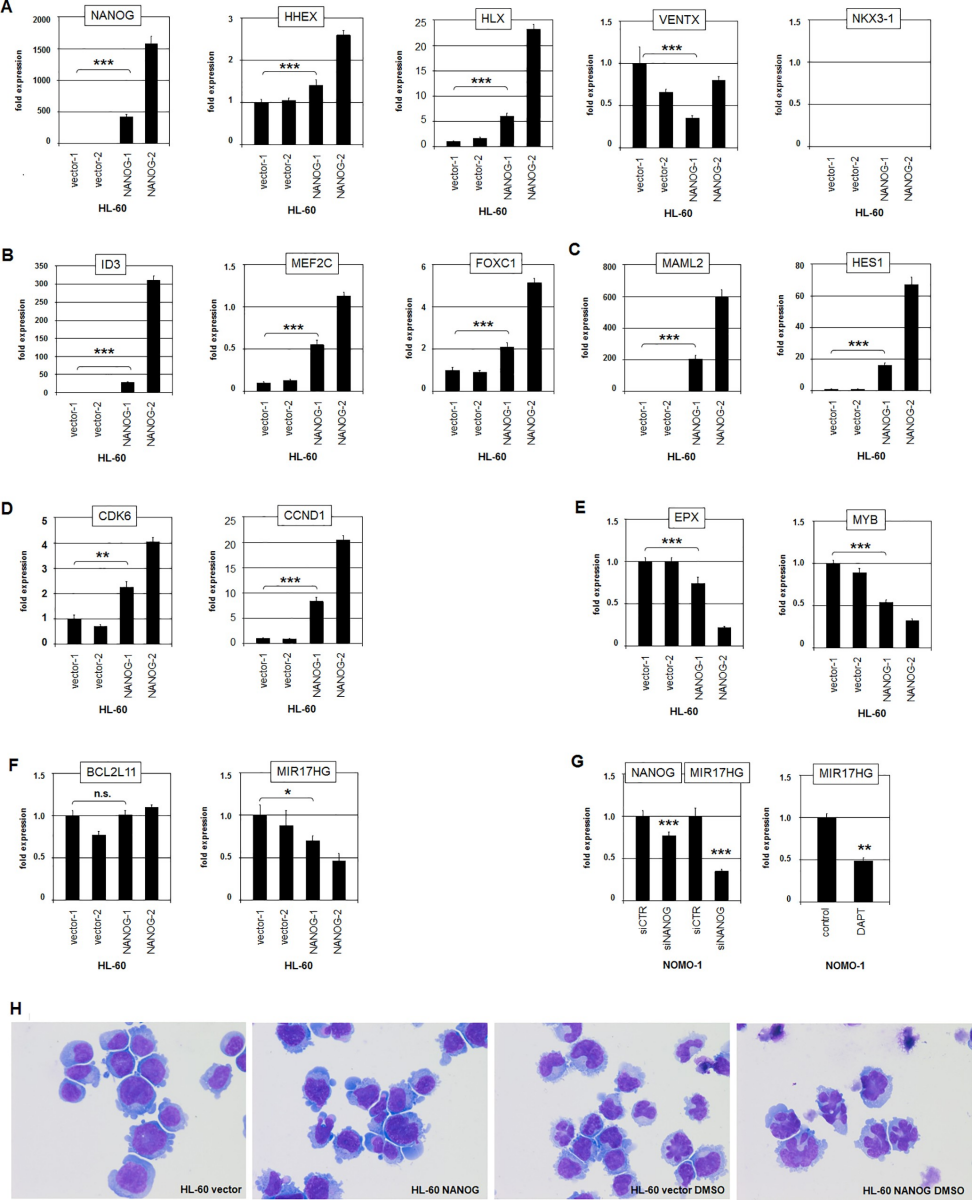
Functional analyses of NANOG in HL-60 cells. HL-60 cells were lentiviral transduced with NANOG (NANOG-1 and NANOG-2) or control vector (vector-1 and vector-2). Subsequent RQ-PCR analyses were performed for selected genes including (A) NKL homeobox genes, (B) transcription factors, (C) NOTCH-pathway factors, (D) proliferation factors, (E) differentiation factors, and (F) apoptosis factors. (G) RQ-PCR analysis of MIR17HG in NOMO-1 cells after siRNA-mediated knockdown of NANOG (left), and after treatment with NOTCH-inhibitor DAPT (right). (H) HL-60 cells were electroporated with NANOG or control vector, treated for 5 days with 1.25% DMSO, and stained with Giemsa.

To search systematically for target genes of NANOG in AML we lentivirally transduced NANOG-low cell line HL-60. Subsequent expression profiling analyses of two polyclonal cell populations overexpressing NANOG and of two vector controls allowed the identification of several differentially expressed genes (**S2 Table**). Gene-annotation enrichment analysis of the top-1000 overexpressed genes in HL-60/NANOG cells revealed GO-terms referring to general signal transduction (p = 6.2x10^-5^) and to the NOTCH-pathway (p = 1.9x10^-2^). Furthermore, visual analysis of the top-1000 differentially overexpressed and downregulated genes indicated several potential target genes, which reportedly perform oncogenic operations. Accordingly, selected candidates were analyzed by RQ-PCR in transduced HL-60 cells, demonstrating that NANOG activated the transcription factors FOXC1, HHEX, HLX, ID3 and MEF2C but inhibited VENTX (**Fig 8A** and **8B**), activated NOTCH-signalling factors MAML2 and HES1 (**Fig 8C**), activated the proliferation factors CCND1 and CDK6 (**Fig 8D**), inhibited differentiation factors EPX and MYB (**Fig 8E**), and inhibited apoptosis-regulator MIR17HG (**Fig 8F**).

Functional analyses of transduced HL-60 cells were performed by live-cell-imaging. HL-60 cells overexpressing NANOG showed no significant change in cell proliferation while etoposide-induced apoptosis was significantly enhanced when compared to controls (**S7B Fig**). HL-60 cells differentiate after treatment with TPA or DMSO, altering their cell morphology into an elongated shape or their nuclei into irregular conformations, respectively. After TPA-stimulation HL-60 cells transfected for overexpression of NANOG showed just slight differences of their shape as compared to the vector control (**S7C Fig**). However, NANOG overexpression induced the generation of nuclei with an irregular conformation, which was further enhanced by DMSO (**Fig 8H**), indicating that NANOG supports cell differentiation in HL-60.

Of note, we observed a substantial loss of NANOG expression in transduced HL-60 cell populations after four weeks of cultivation, matching its observed negative impact on cell survival and its positive impact on cell differentiation. Taken together, this overexpression-approach in HL-60 AML cells confirmed HHEX, VENTX and the NOTCH-pathway as downstream targets of NANOG as shown above in NOMO-1. Moreover, we identified additional transcription factors, cell cycle regulators and differentiation markers deregulated by NANOG. Repression of anti-apoptosis gene MIR17HG may underlie the enhanced sensitivity for etoposide-induced apoptosis in NANOG-positive HL-60 cells [46,47].

### Deregulation of MIR17HG in myeloid malignancies

In contrast to NANOG-mediated repression of MIR17HG in HL-60 our gene expression data have shown that AML cell line NOMO-1 shared elevated transcript levels of MIR17HG with chronic myeloid leukemia (CML) cell line K-562 (**Fig 5B**). Interestingly, copy number gains at 13q31 comprising the MIR17HG locus, were detected in both cell lines and may thus underlie MIR17HG activation (**S9A Fig**). However, this gain was much stronger in K-562 and matched those of the loci of BCR at 22q11 and ABL1 at 9q34 (**S9A Fig**). Furthermore, FISH analysis of K-562 demonstrated co-amplification of the fusion gene BCR-ABL1 together with the micro-RNA gene cluster MIR17HG (**S9B Fig**). Intriguingly, MIR17HG represents a target gene of BCR-ABL1 [29]. Thus, co-amplification of both genes contributed to an enhanced expression level of this micro-RNA encoding gene in CML cells.

In NOMO-1 siRNA-mediated knockdown of NANOG resulted in reduced expression of MIR17HG (**Fig 8G**), demonstrating that its reported potential to transactivate this anti-apoptosis factor operated in AML as well [48]. Consistently, treatment of NOMO-1 with NOTCH-inhibitor DAPT also resulted in reduced expression of MIR17HG (**Fig 8G**), highlighting a role of the NOTCH-NANOG-axis in MIR17HG regulation. Moreover, etoposide-induced apoptosis in NOMO-1 was further increased by treatment with DAPT (**S7D Fig**), demonstrating anti-apoptotic activity of NOTCH and NANOG. Thus, elevated expression levels of MIR17HG correlated in NOMO-1 and K-562 with copy number gains. In addition, NOMO-1 expressed NANOG and K-562 fusion protein BCR-ABL1 to enhance MIR17HG expression and to suppress apoptosis.

## Discussion

In this study we analyzed the expression of NKL homeobox genes in normal developing and mature myeloid cells, generating a six gene strong myeloid NKL-code. The extended hematopoietic NKL-code now consists of eleven genes comprising DLX2, HHEX, HLX, HMX1, MSX1, NANOG, NKX2-3, NKX3-1, NKX6-3, TLX2 and VENTX. Our key observations concerning the activity of NKL-code members were as follows: (1) HHEX, HLX, NKX3-1 and VENTX represent the most prominent myeloid NKL homeobox genes; (2) differential levels of these four genes generated subtype-specific patterns in granulocytes; (3) DLX2 expression was restricted to monocytes, mast cells and dendritic cells; (4) HMX1 was specific for the erythropoiesis; (5) strikingly high expression levels of NKX3-1 were detected in monocytes and of HLX in neutrophils and monocytes; (6) monocyte-derived macrophages and dendritic cells showed altered expression levels of DLX2, HHEX, HLX, NKX3-1 and VENTX when compared to monocytes. These gene signatures indicated specific functions for particular NKL subclass members in myeloid cell differentiation. Of note, STAT3-activation is responsible for the differentiation of both neutrophils and monocytes/macrophages [49–53]. These data together with the reported potential of STAT3 to activate HLX expression in lymphoid cells supported the conclusion that elevated HLX levels as observed here in neutrophils and monocytes also reflect STAT3-activation [43,44].

The number of identified NKL homeobox genes deregulated in AML and MDS resembled those described in lymphoid malignancies including T/B/NK-cell leukemia/lymphoma [12–14,17]. This observation supports the significance of this group of developmental regulators for hematopoietic malignancies in general. However, no or few aberrantly overexpressed NKL homeobox genes have been detected in cell lines derived from primary effusion lymphoma and multiple myeloma, respectively, representing mature B-cell lymphomas [27]. Concomitant downregulation of NKL-code members rather indicated tumor suppressor functions for NKL homeobox genes in these malignancies.

As reported so far, aberrantly expressed NKL homeobox genes in AML include DLX1 and DLX2 [18], HHEX [54,55], HLX [19], MSX1 [56], NANOG [57], and VENTX [20,58]. Our study added several other NKL subclass members to this list. Accordingly, we have detected the ectopic activity of DLX5 and DLX6 in AML cell line NOMO-1 and of DLX6 in AML patients. These genes are normally silent in the hematopoietic system and represent paradigms for deregulated NKL homeobox genes in myeloid leukemia, which are physiologically implicated in the development of neural crest cells. This relationship has also been shown for MSX1 and NKX6-3 in lymphoid leukemia/lymphoma [13,36,59–61]. Of note, we failed to detect aberrant expression of TLX1/2/3 in AML or MDS. Thus, TLX genes may represent hallmark oncogenes for lymphoid T-cell leukemia [62–64]. Nevertheless, due to the structural similarity of NKL homeobox genes we propose that they share oncogenic functions and may thus serve as therapeutic targets in hematopoietic tumors. To reveal such general activities, it is reasonable to study aberrantly expressed NKL homeobox genes in more detail and to compare their activities.

**Fig 9:**
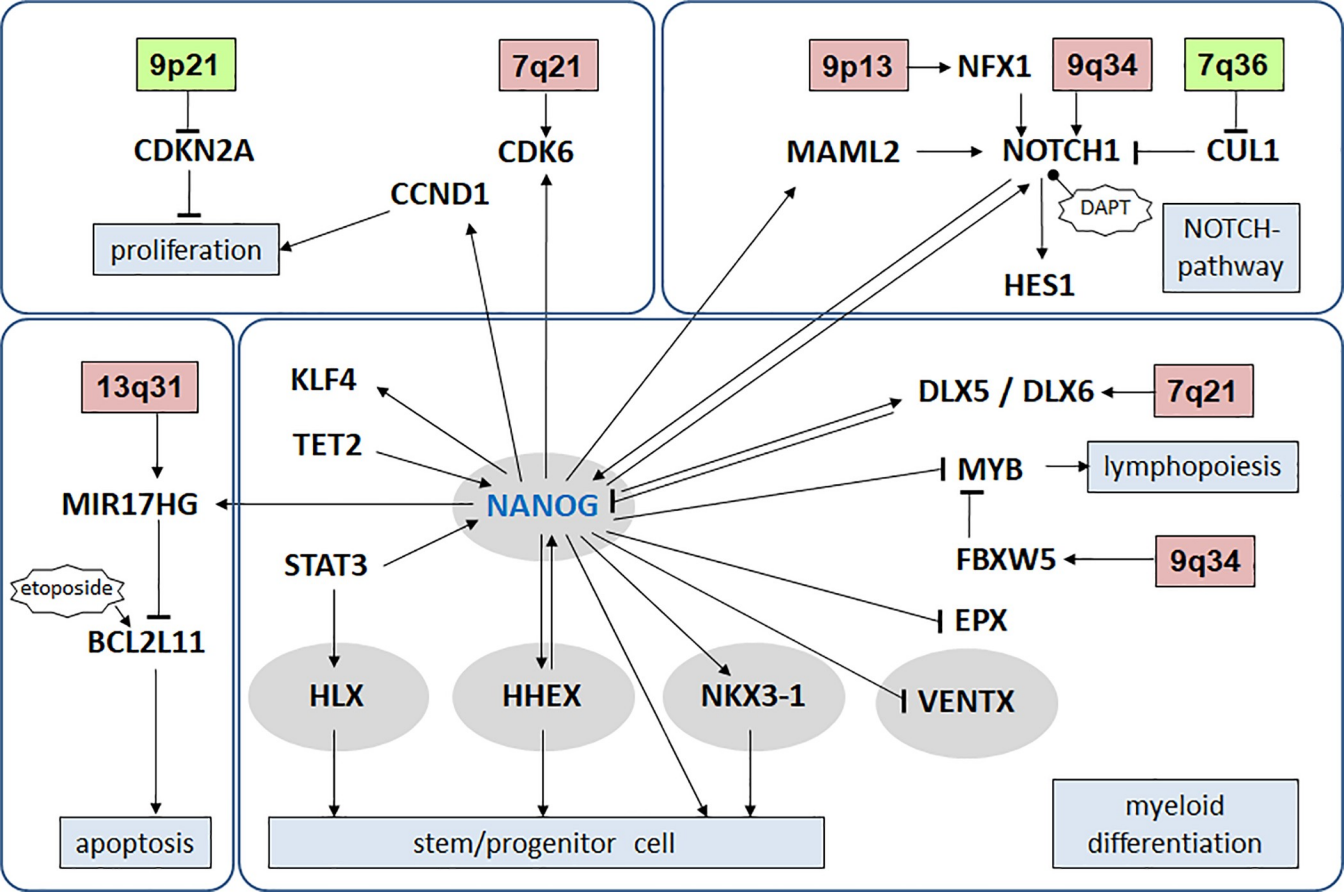
Overview of NANOG in a regulatory network. NANOG in addition to HLX, HHEX, NKX3-1 and VENTX are members of the hematopoietic NKL-code and are highlighted by blue ovals. NANOG is located in the middle of a network, which regulates several basic processes including proliferation, NOTCH-signalling, apoptosis and differentiation. Genomic loci targeted by copy number changes in NOMO-1 are indicated.

Therefore, we focused here on deregulated NKL homeobox gene NANOG in AML analyzing patients and cell lines. Our data places NANOG at a central position in a myeloid gene regulatory network (**Fig 9**), containing NKL-code genes HHEX, HLX, NKX3-1 and VENTX, in addition to non-hematopoietic NKL homeobox genes DLX5 and DLX6. Thus, NANOG belongs to the NKL-code and impacts other code-members. In addition, NANOG suppressed known hematopoietic differentiation genes including peroxidase EPX and transcription factor MYB. Moreover, elevated expression levels of the proteasomal factor FBXW5 may contribute to the downregulation of MYB as well [65]. NANOG may act as a differentiation factor in early hematopoiesis and as an oncogene in a small subset of AML. In several solid cancer types NANOG has been described as an oncogene as well [66]. NANOG generates clusters of binding in ESCs and impacts the genome organization, which may underlie its oncogenic activity to deregulate downstream genes [67,68]. Observed context-dependent activities of target genes may be thus explained by differences in cofactors and chromatin structures.

The expression of NANOG itself has been correlated with the leukemic fusion protein KMT2A-MLLT2 [35]. However, cell line MV4-11 contains KMT2A-MLLT2 but does not express elevated NANOG, discounting this fusion protein as major activator in AML. Moreover, our data excluded KMT2A and thus fusion protein KMT2A-MLLT3 from NANOG activation in NOMO-1. Consistently, NANOG expression showed no even level in AML patients carrying translocations of KMT2A (**S5** and **S10 Figs**). But in HL-60 NANOG activated the expression of transcription factor FOXC1 as described recently in AML patients and was additionally associated in NOMO-1 with FLT3-deregulation, which may play a supporting role in leukemogenesis as well [33,69].

We detected regulatory connections between NANOG and the NOTCH-pathway by diverse methods including comparative expression profiling, knockdown of NANOG in NOMO-1, and its overexpression in HL-60. Furthermore, copy number analysis in NOMO-1 cells revealed genomic gains of NOTCH1 and NFX1, and a genomic loss of CUL1. NFX1 activates NOTCH1 and may contribute to NANOG expression via its downstream activity [70]. CUL1 mediates proteasomal degradation of NOTCH1, and MAML2—activated by NANOG in HL-60 cells—acts as an activator of NOTCH-signalling [71,72]. Finally, DLX5 activates NOTCH signalling in malignant T-cells and uremic kidney [73,74], suggesting that this aberrantly expressed NKL homeobox gene may regulate NANOG expression via this pathway in NOMO-1 as well. However, the NOTCH-pathway plays a controversial role in AML, performing context-dependent either tumor suppressor or oncogenic functions [38].

Our data indicated that NANOG regulates apoptosis via MIR17HG. This gene encodes six different micro-RNAs and has been reported as a target gene of several other oncogenic members of the NKL homeobox gene subclass [46,75]. In CML MIR17HG is activated by fusion protein BCR-ABL1 regulating apoptosis via BCL2 and BCL2L11 [29]. The presence of fusion genes BCR-ABL1 and NUP214-XKR3 in CML cell line K-562 suggests an early and simultaneous generation of both translocations while juxtaposition and subsequent co-amplification of BCR-ABL1 and MIR17HG as reported here may have occurred afterwards (**S9C Fig**) [76]. In cell line NOMO-1 NANOG activated the expression of MIR17HG, which exhibited amplification as well. Forced expression of NANOG in additional AML cell lines (GF-D8, ME-1, MV4-11) resulted in elevated transcription of MIR17HG as well (**S9D Fig**), indicating a frequent activatory connection in this malignancy. In cell line HL-60 NANOG repressed the expression of MIR17HG, performed pro-apoptotic effects, and induced cell differentiation. Thus, HL-60 and NOMO-1 represent differing models to analyze functional aspects of NANOG in AML. These results indicated that in particular myeloid contexts NANOG acts as tumor suppressor instead of an oncogene. But due to its reported impacts in self-renewal and stemness NANOG-positive cases may deserve special therapeutic attention.

NKL homeobox gene NANOG is normally only active in early hematopoietic cell differentiation, probably influencing the stem cell status and the development of both lymphopoiesis and myelopoiesis [12]. Here, we analyzed the relationship between NANOG and the stem cell factors STAT3, TET2, TP63 and KLF4. STAT3 activates transcription of NANOG in ESCs directly [42]. In NOMO-1 cells STAT3 activated NANOG as well, possibily recapitulating this regulatory connection. In ESCs the NOTCH-target HES1 activates STAT3 [77]. This impact may also play a role in AML as forced expression of NANOG in HL-60 cells resulted in enhanced HES1 activity.

Furthermore, global mapping of DNA-methylation has revealed hypomethylation of the murine NANOG promoter in ESCs and hypermethylation in differentiated cells [78]. Accordingly, NANOG is regulated by promoter-methylation in human ESCs and lymphocytes, and treatment of murine ESCs with AZA resulted in increased expression of NANOG [79,80]. TET2 is a DNA-demethylase, involved in stem cell regulation, and binds to the promoter-region of NANOG in human and murine ESCs [45,81,82]. Consistently, we identified overexpression of TET2 in NOMO-1, which activated the expression of NANOG. In this cell line TET2 acts as an oncogene while in other AML cases TET2 is mutated and performs tumor suppressor activity [83,84]. Stem cell factor TP63 has been associated with AML but is not regulatory connected with NANOG [85,86].

In NOMO-1 NANOG activated KLF4 but KLF4 did not influence the expression of NANOG in return. This observation contrasts ESCs in which NANOG activates KLF4 transcription and interacts with the encoded protein for stabilization [87]. KLF4 performs both stem cell maintenance and monocyte differentiation and may thus play dual roles in AML cells [40,88]. Of note, in the latter context KLF4 has been described to supply tumor suppressor functions [89]. Thus, we identified context-dependent activities of NANOG in relation to KLF4, as well as NOTCH-signalling and MIR17HG. These circumstances may explain the dearth of NANOG deregulated in AML.

In conclusion, we presented a physiological expression pattern for NKL homeobox genes covering the myeloid system, which extended the reported hematopoietic NKL-code. We identified several deregulated NKL homeobox genes in AML and MDS, highlighting the significance of these genes for myeloid malignancies, which may serve as diagnostic markers and rational therapeutic targets in the future. NANOG is only rarely expressed in subsets of AML, which may reflect context dependent functions such as the regulation of MIR17HG expression and cell differentiation, thus confirming NANOG´s status as a fundamental oncogene in AML.

## Supporting information

S1 Fig Screening results for NKL homeobox genes in normal myelopoiesis. (TIF)

S2 Fig Screening results for NKL homeobox genes in AML patients. (TIF)

S3 Fig Screening results for NKL homeobox genes in MDS patients. (TIF)

S4 Fig Screening results for NKL homeobox genes in AML cell lines. The highest levels of NANOG are expressed by GDM-1 (446735), PL-21 (446753), and NOMO-1 (446755).
(TIF)

S5 Fig Expression profiling data for NANOG in AML patients. (TIF)

S6 Fig **Comparative expression profiling analysis of (A) AML patients, and (B) AML cell lines.** NANOG-high cell lines used for comparative analyses are GDM-1 (446735), PL-21 (446753), and NOMO-1 (446755); NANOG-low control cell lines are EOL-1 (446733), HL-60 (446736), KASUMI-1 (446745), MV4-11 (446746), KG-1A (446747), and GF-D8 (446759).
(TIF)

S7 Fig Life-cell-imaging and cell differentiation results. (A) NOMO1 cells treated with NOTCH-inhibitor DAPT were analyzed for proliferation (left) and apoptosis (right). (B) Transduced HL-60/NANOG cells treated with etoposide were analyzed for proliferation (left) and apoptosis (right). (C) Treatment of HL-60 cells with TPA induced an elongated cell shape as documented by microscopic pictures taken by the IncuCyte system after 24 h (right). Normal HL-60 cells (middle) and transfected HL-60 cells (right) were analyzed for morphological eccentricity. (D) NOMO1 cells treated with NOTCH-inhibitor DAPT in combination with etoposide were analyzed for apoptosis.
(TIF)

S8 Fig RNA-seq data for myeloid cell lines. (A) Expression data of OSKM-factors. (B) Expression data of DNA-methylation-related genes. Arrows indicate NOMO-1.
(TIF)

S9 Fig MIR17HG—Genomic profiling, FISH analysis and expression. (A) Genomic profiling data of K-562 and NOMO-1 for chromosomes 13, 22, and 9. (B) FISH analysis of K-562 using probes for MIR17HG (red), BCR (yellow), and ABL1 (green), demonstrating co-amplification. Chromosomes were counterstained with DAPI (blue). (C) Focal genomic profiling data of K-562 chromosome 22 (above) and chromosome 9 (below), showing loci implicated in the generation of fusion genes. (D) RQ-PCR analysis of MIR17HG expression in MV4-11 (left), GF-D8 (middle) and ME-1 (right) after transfection of NANOG.
(TIF)

S10 Fig NANOG expression in AML patients. Dataset GSE19577 contains 42 AML patients with different KMT2A-translocations. The expression values of NANOG show varying levels indicating independent activation mechanisms.
(TIF)

S1 Table Combined analysis of genome and transcriptome data. (XLSX)

S2 Table Expression profiling data of HL-60/NANOG. (XLS)
