# Supplementary figures and images for: NKL homeobox gene activities in normal and malignant myeloid cells
Source: PLoS One. 2019 Dec 11;14(12):e0226212. doi: 10.1371/journal.pone.0226212 (PMC6905564; doi:10.1371/journal.pone.0226212)

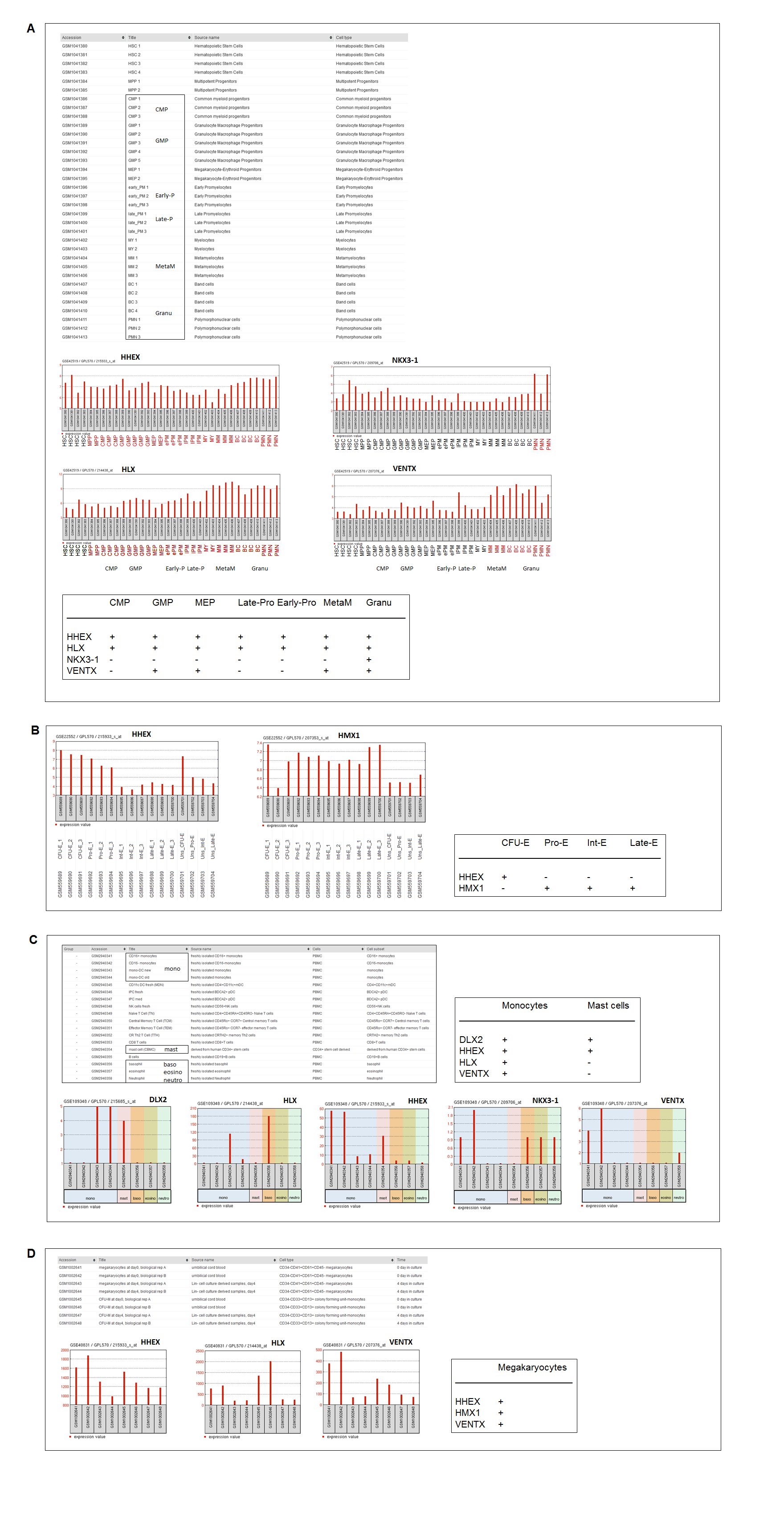

Supplement: S1 Fig — (TIF) [file pone.0226212.s001.tif]

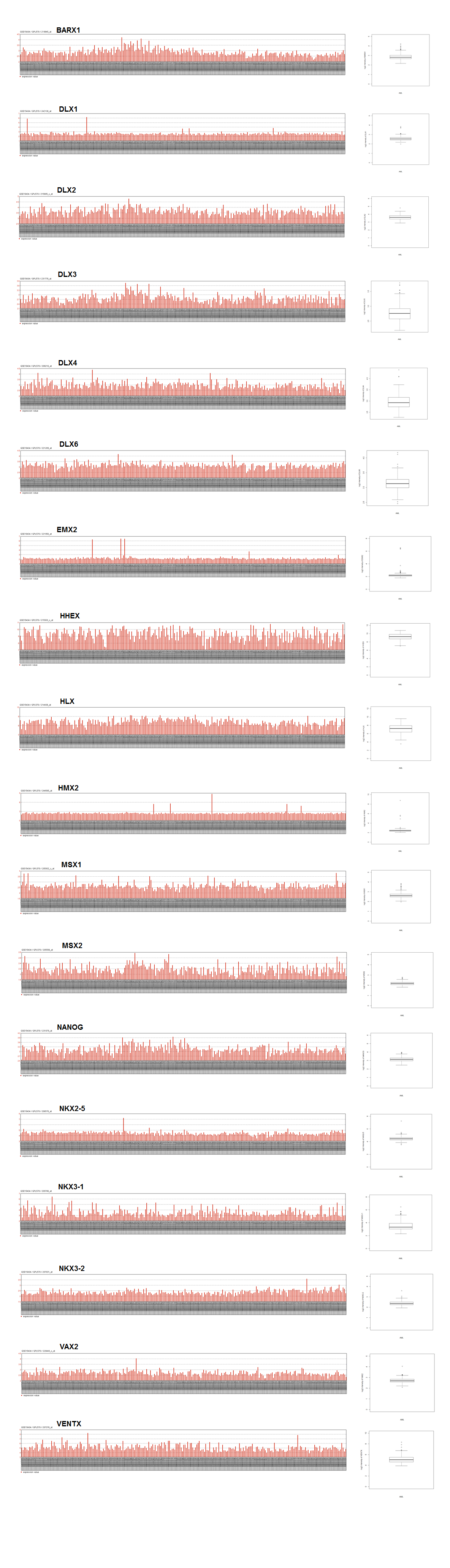

Supplement: S2 Fig — (TIF) [file pone.0226212.s002.tif]

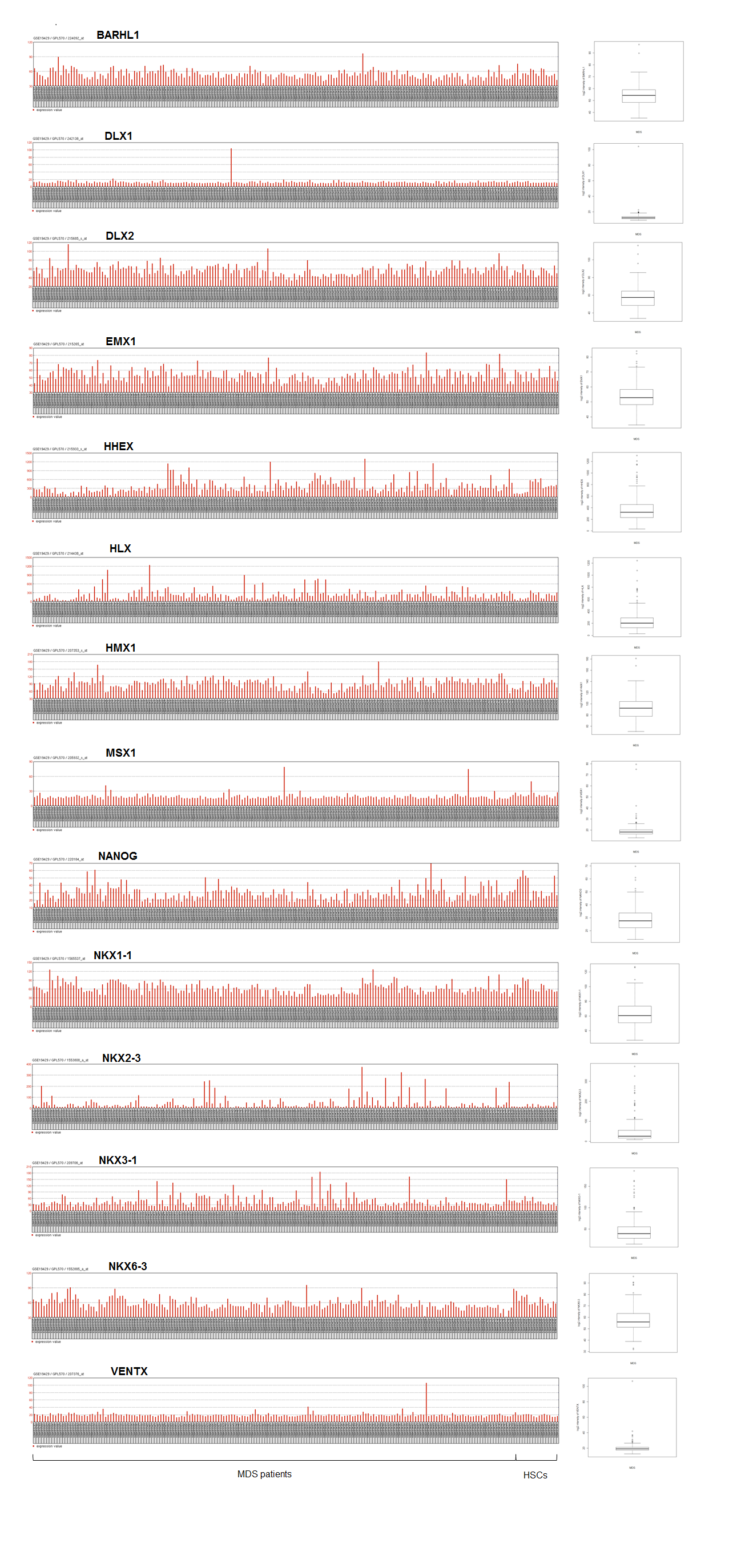

Supplement: S3 Fig — (TIF) [file pone.0226212.s003.tif]

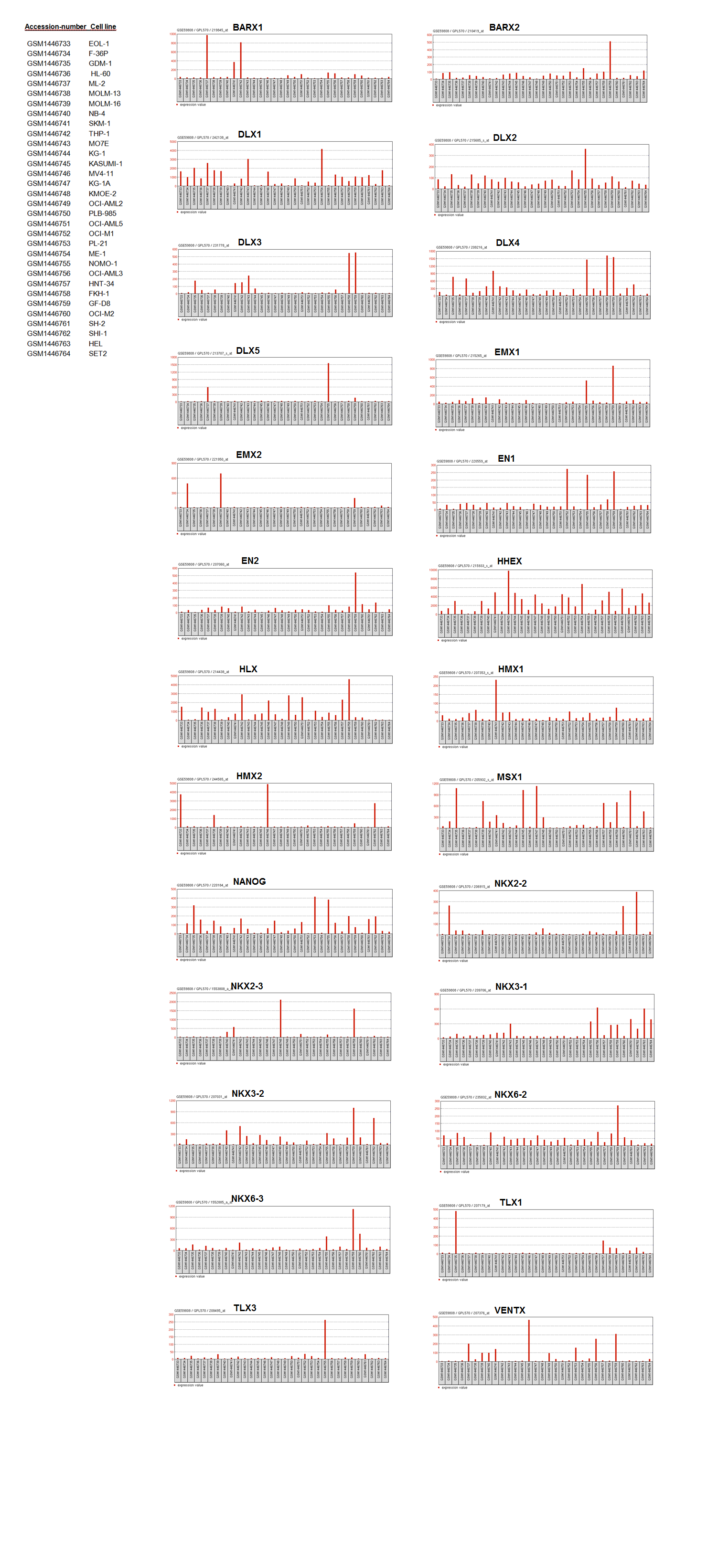

Supplement: S4 Fig — The highest levels of NANOG are expressed by GDM-1 (446735), PL-21 (446753), and NOMO-1 (446755). (TIF) [file pone.0226212.s004.tif]

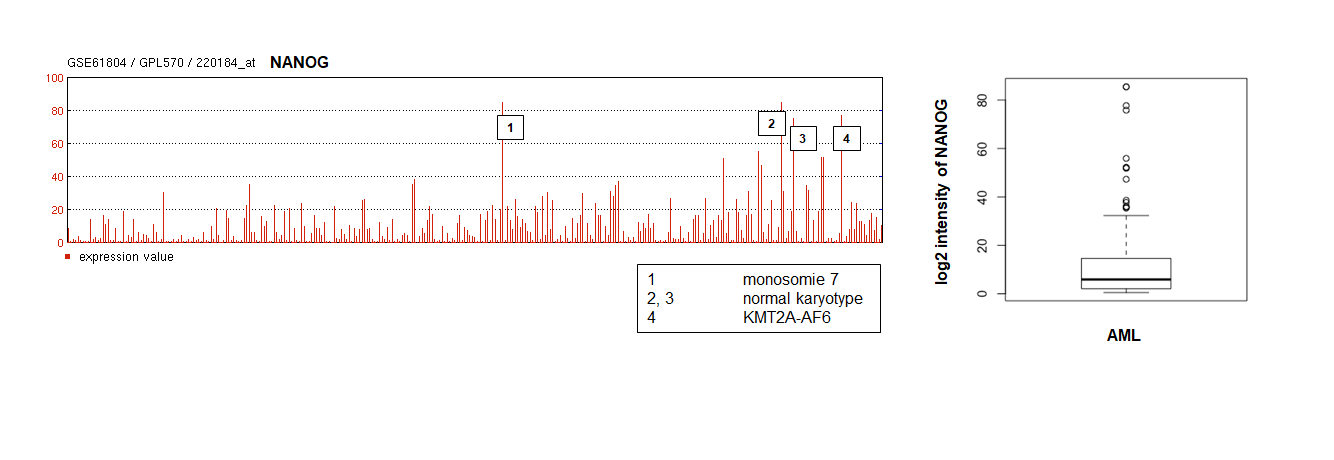

Supplement: S5 Fig — (TIF) [file pone.0226212.s005.tif]

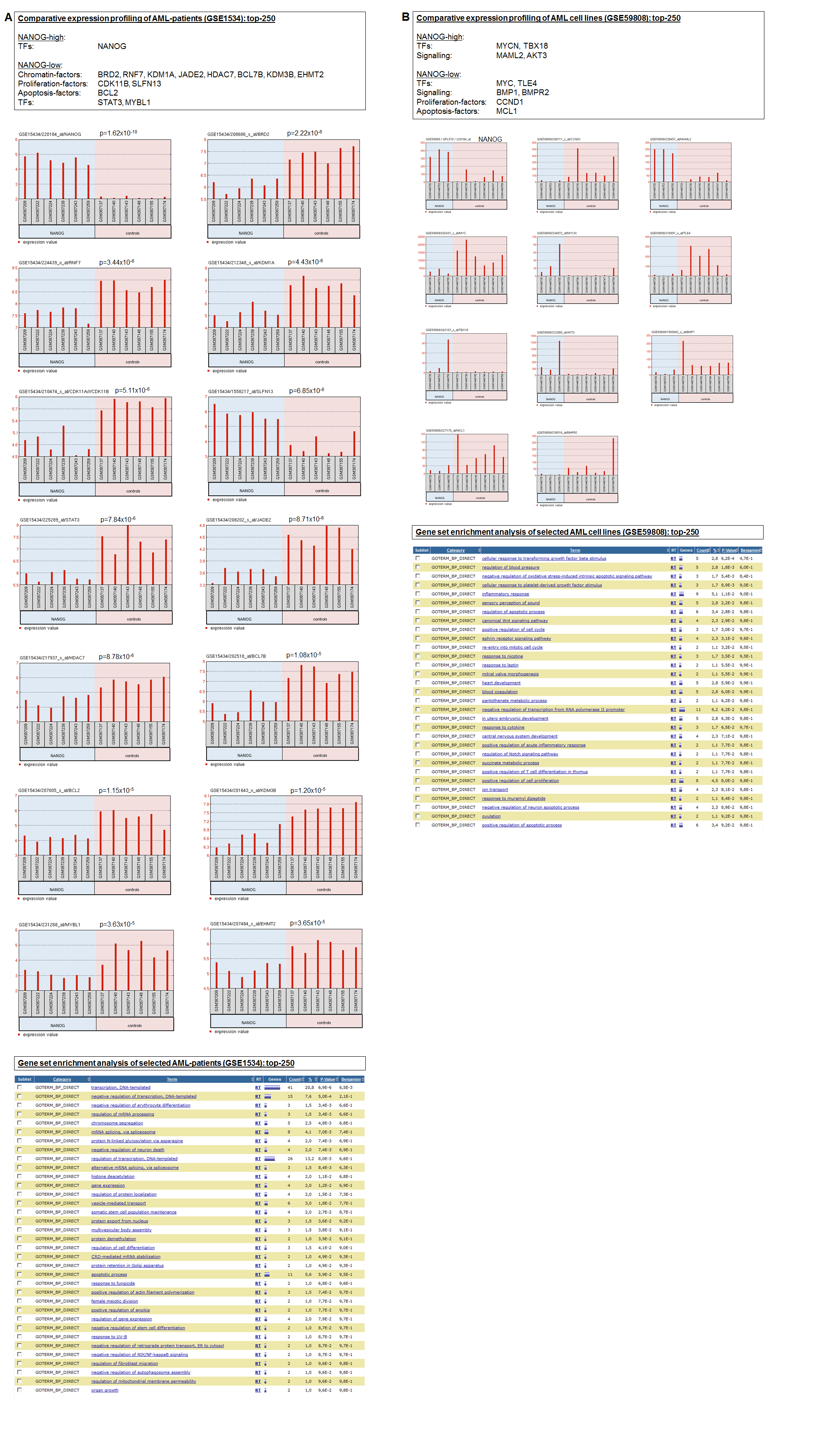

Supplement: S6 Fig — Comparative expression profiling analysis of (A) AML patients, and (B) AML cell lines. NANOG-high cell lines used for comparative analyses are GDM-1 (446735), PL-21 (446753), and NOMO-1 (446755); NANOG-low control cell lines are EOL-1 (446733), HL-60 (446736), KASUMI-1 (446745), MV4-11 (446746), KG-1A (446747), and GF-D8 (446759). (TIF) [file pone.0226212.s006.tif]

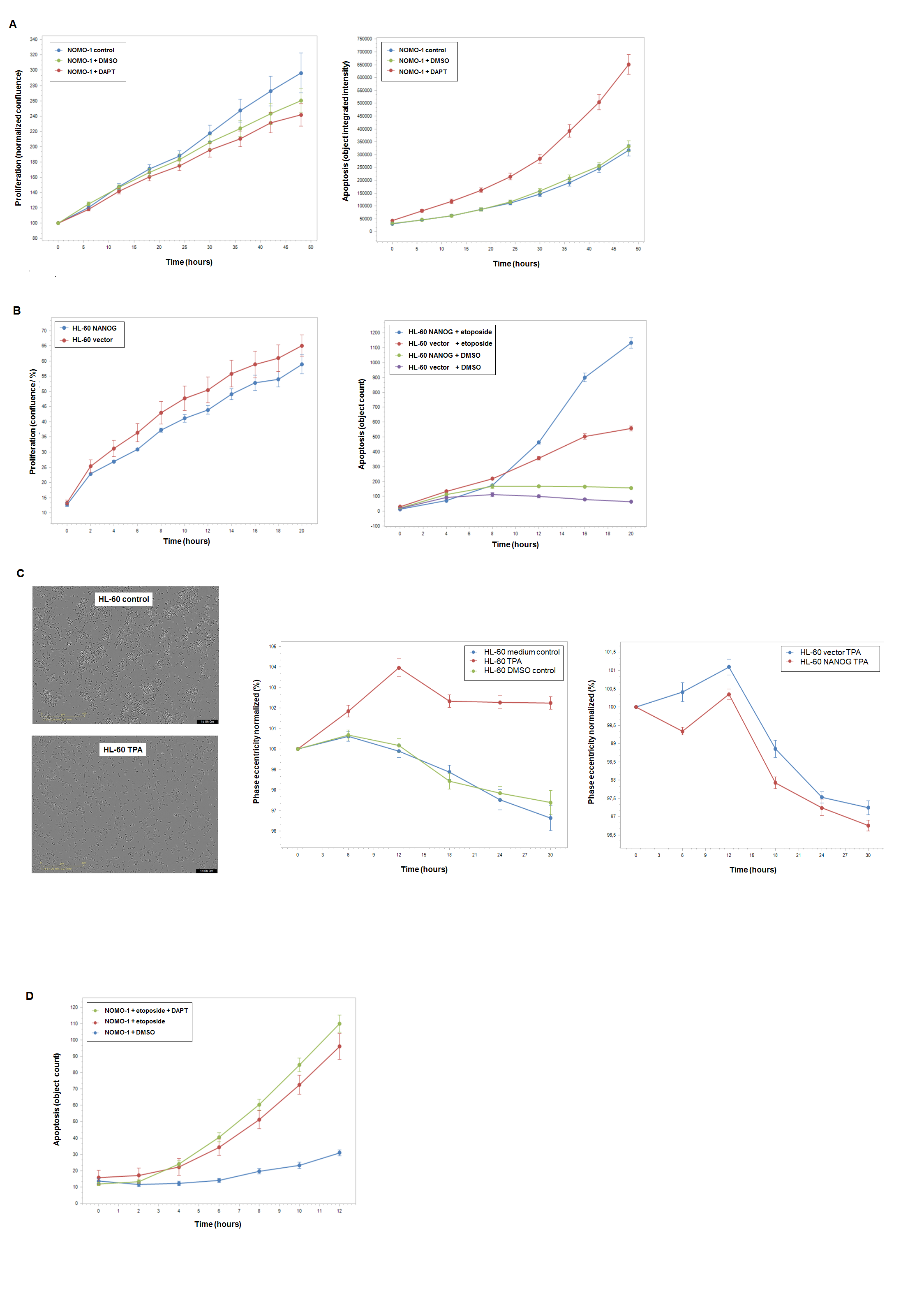

Supplement: S7 Fig — (A) NOMO1 cells treated with NOTCH-inhibitor DAPT were analyzed for proliferation (left) and apoptosis (right). (B) Transduced HL-60/NANOG cells treated with etoposide were analyzed for proliferation (left) and apoptosis (right). (C) Treatment of HL-60 cells with TPA induced an elongated cell shape as documented by microscopic pictures taken by the IncuCyte system after 24 h (right). Normal HL-60 cells (middle) and transfected HL-60 cells (right) were analyzed for morphological eccentricity. (D) NOMO1 cells treated with NOTCH-inhibitor DAPT in combination with etoposide were analyzed for apoptosis. (TIF) [file pone.0226212.s007.tif]

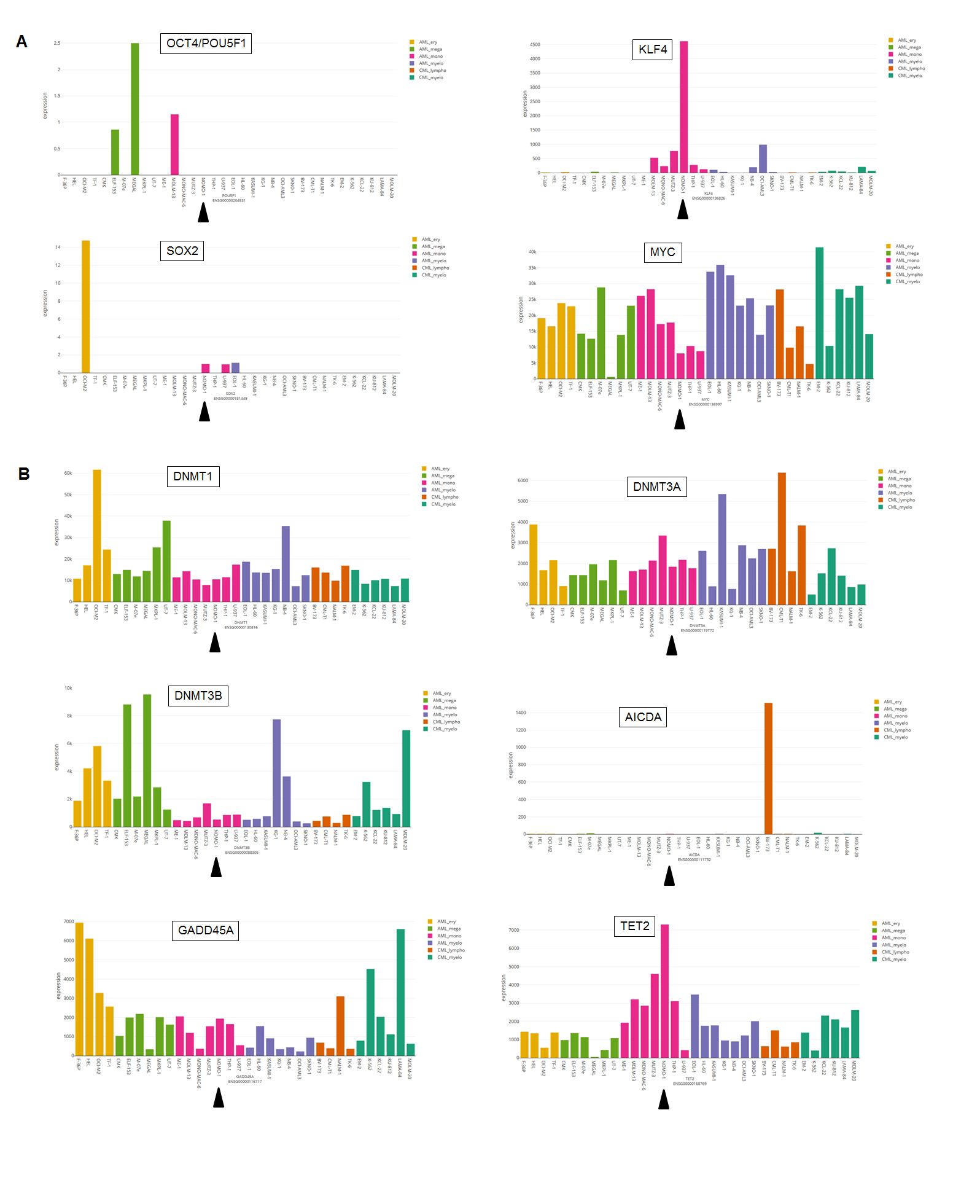

Supplement: S8 Fig — (A) Expression data of OSKM-factors. (B) Expression data of DNA-methylation-related genes. Arrows indicate NOMO-1. (TIF) [file pone.0226212.s008.tif]

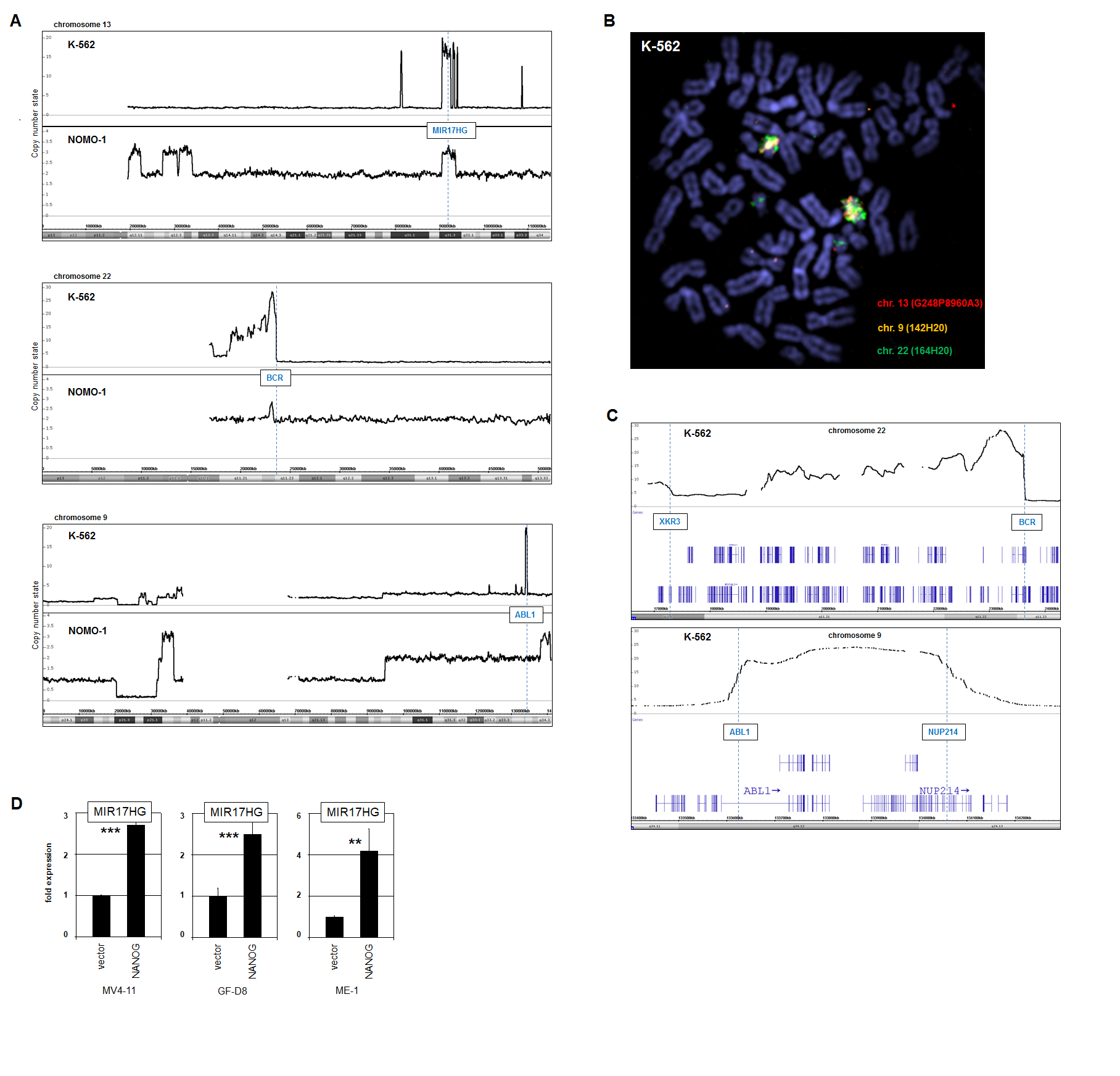

Supplement: S9 Fig — (A) Genomic profiling data of K-562 and NOMO-1 for chromosomes 13, 22, and 9. (B) FISH analysis of K-562 using probes for MIR17HG (red), BCR (yellow), and ABL1 (green), demonstrating co-amplification. Chromosomes were counterstained with DAPI (blue). (C) Focal genomic profiling data of K-562 chromosome 22 (above) and chromosome 9 (below), showing loci implicated in the generation of fusion genes. (D) RQ-PCR analysis of MIR17HG expression in MV4-11 (left), GF-D8 (middle) and ME-1 (right) after transfection of NANOG. (TIF) [file pone.0226212.s009.tif]

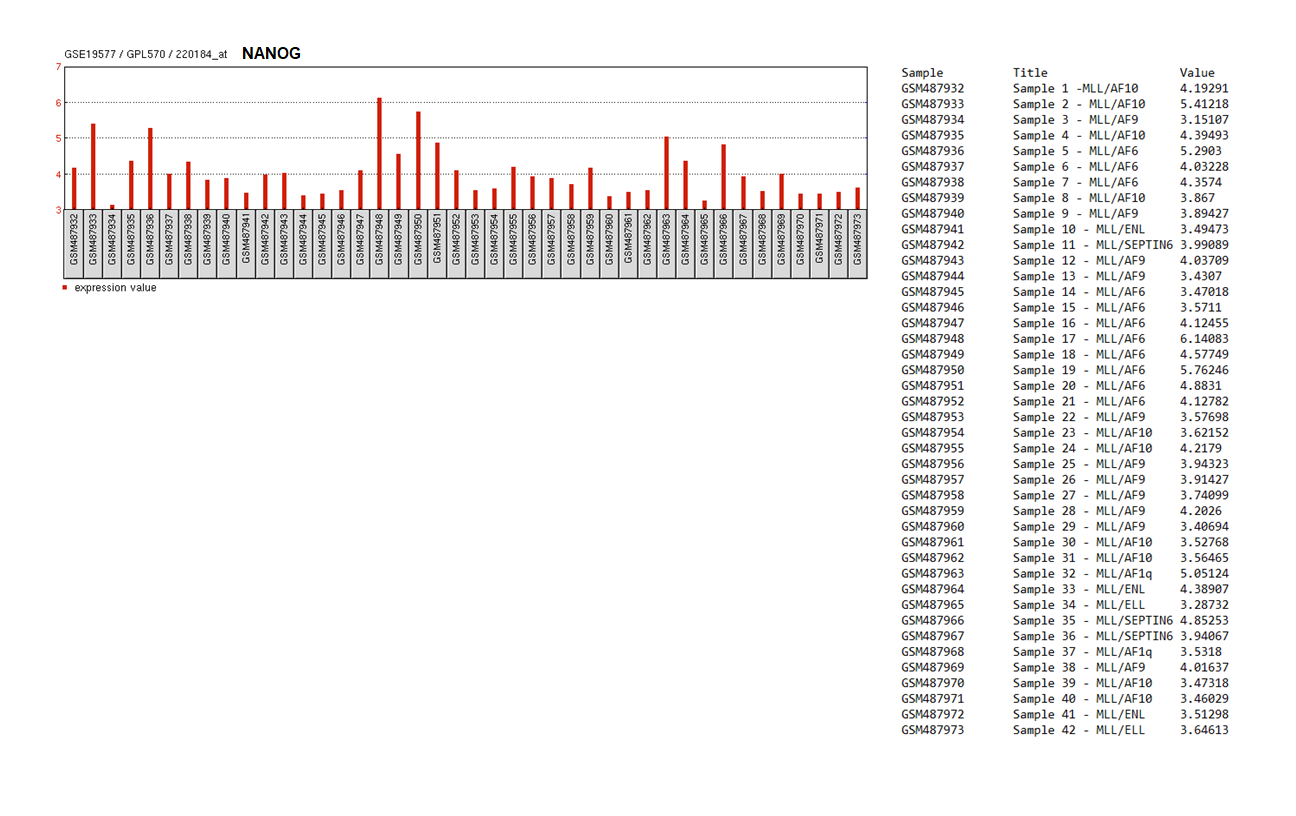

Supplement: S10 Fig — Dataset GSE19577 contains 42 AML patients with different KMT2A-translocations. The expression values of NANOG show varying levels indicating independent activation mechanisms. (TIF) [file pone.0226212.s010.tif]
